# TET1 loss propels the development of hyperthyroidism by remodeling histone modifications of *PAX8* promoter

**DOI:** 10.1038/s12276-025-01566-2

**Published:** 2025-10-29

**Authors:** Hui Dang, Yan Liu, Ye Zhou, Mengjun Sui, Yubo Wang, Wei Qiang, Fang Sui, Yan Zhang, Hongxin Cao, Xiaoyan Wu, Meiju Ji, Peng Hou

**Affiliations:** 1 Department of Endocrinology and Metabolism, The First Affiliated Hospital of Xi’an Jiaotong University, Xi’an, People’s Republic of China; 2 Department of Otorhinolaryngology-Head and Neck Surgery, The First Affiliated Hospital of Xi’an Jiaotong University, Xi’an, People’s Republic of China; 3 Center for Translational Medicine, The First Affiliated Hospital of Xi’an Jiaotong University, Xi’an, People’s Republic of China

**Keywords:** Endocrine system and metabolic diseases, Molecular biology

## Abstract

Ten eleven translocation 1 (TET1) is a 5-methylcytosine dioxygenase, and its altered DNA demethylation has been implicated in human diseases. However, its role in regulating thyroid function remains totally unknown. Here we first generated thyroid-specific *Tet1* knockout combined with thyroid-specific *Braf*^*V600E*^ transgenic mouse model (*Thy-Braf*^*V600E*^*; Tet1*^*−/−*^) and their control mice (*Thy-Braf*^*V600E*^*; Tet1*^*+/+*^). The latter developed severe hypothyroidism and lost reproductive ability owing to structural damages of thyroid gland, while thyroid-specific *Tet1* knockout effectively restored thyroid structure and function of *Thy-Braf*^*V600E*^*; Tet1*^*+/+*^ mice and their reproductive ability. In addition, we also established thyroid-specific *Tet1* knockout mouse model (*Thy-Tet1*^*−/−*^) and demonstrated that these mice could develop hyperthyroidism with systemic hypermetabolic symptoms such as weight loss, increased heart rate and elevated systolic blood pressure, further supporting the inhibitory effect of TET1 on thyroid function. Transcriptomic sequencing revealed that key genes related to metabolism and synthesis of thyroid hormones such as *PAX8*, *SLC5A5* and *TPO* were significantly upregulated in *Thy-Tet1*^*−/−*^ mice. Mechanistically, TET1 recruits HDAC1 to reduce the levels of H3K27Ac and H3K9Ac in the *PAX8* promoter, thereby inhibiting the expression of itself and its downstream targets NIS and TPO. Further studies showed that elevated miR-29c-3p in serum exosomes enhanced thyroid function by targeting TET1, which may be one of the causes of hyperthyroidism. Thus, this study uncovers a new mechanism by which TET1 suppresses thyroid function, providing a new perspective to explore the pathogenesis of hyperthyroidism.

## TET1 regulates thyroid function and hyperthyroidism mechanisms

Hyperthyroidism is a condition in which the thyroid gland produces too many hormones, leading to symptoms such as weight loss and irritability. Here scientists are exploring the role of a protein called TET1 in thyroid function. TET1 is known for its role in modifying DNA, which can affect how genes are turned on or off. In this study, researchers investigated whether TET1 influences thyroid activity. They used mice that were genetically modified to lack TET1 specifically in their thyroid glands. These mice showed signs of hyperthyroidism such as increased thyroid hormone levels and faster metabolism. The researchers found that TET1 normally helps suppress the activity of certain genes involved in thyroid hormone production by interacting with other proteins that modify DNA structure. This study suggests that TET1 plays a crucial role in regulating thyroid function and that its absence can lead to hyperthyroidism.

This summary was initially drafted using artificial intelligence, then revised and fact-checked by the author.

## Introduction

Hyperthyroidism is a pathological disorder caused by the hyperfunction of thyroid gland itself, resulting in excessive synthesis and secretion of thyroid hormones^1^. In addition to abnormal thyroid hormone levels, the characteristic physical findings are weight loss, tachycardia and emotional irritability, which are consistent with the clinical features of thyrotoxicosis^1^. The escalating prevalence of hyperthyroidism has caused a growing burden on society in recent years^2,3^. The common pathogenesis and mechanisms of hyperthyroidism include various conditions, such as Graves’ disease, toxic multinodular goiter and toxic adenoma^4,5^. However, the role of additional molecular mechanisms remains unknown, particularly whether epigenetic modifications are involved.

DNA methylation and demethylation are important ways of epigenetic modification, which are involved in regulating gene expression in multiple biological processes and diseases by interacting with transcription factors or altering the structure of chromatin^6^. Ten eleven translocation 1 (TET1), as a DNA dioxygenase, binds to CpG enriched sites and orchestrates the active demethylation process of cytosine by sequentially oxidizing 5-methylcytosine (5mC) to 5-hydroxymethylcytosine (5hmC), which plays an essential role in numerous human diseases^7^. In addition, TET1 also exhibits nonenzymatic activity that recruits or repels other proteins, thereby participating in the chromatin remodeling processes^8^. Functional studies of TET1 have predominantly focused on tumorigenesis, embryonic stem cell differentiation and neurological disorders^9–11^. However, its role in metabolic diseases, especially in thyroid dysfunction, is rarely reported. Histone acetylation is another important epigenetic modification that changes chromatin architecture and regulates gene expression by opening or closing the chromatin structure, which can be dynamically regulated by histone acetyltransferases and histone deacetylases (HDACs) ^12^. There is increasing evidence showing that the dysregulation of histone acetylation contributes to various human diseases, including metabolic diseases^12–14^.

Previous studies have shown that the thyroid-specific *Braf*^*V600E*^ transgenic mouse model (*Thy-Braf*^*V600E*^) spontaneously developed papillary thyroid carcinoma, accompanied by severe hypothyroidism and lost reproductive ability due to structural damages of thyroid gland^15^. In the present study, we unexpectedly discovered that thyroid-specific *Tet1* knockout markedly restored thyroid function and reproductive ability of *Thy-Braf*^*V600E*^ mice. Moreover, we also demonstrated that TET1 was significantly downregulated in thyroid tissues from patients with hyperthyroidism compared with healthy controls, suggesting that TET1 negatively regulates thyroid function. As supported, we established thyroid-specific *Tet1* knockout mouse model (*Thy-Tet1*^*−/−*^) and demonstrated that these mice could develop hyperthyroidism with systemic hypermetabolic symptoms, further supporting the above conclusion. A further mechanistic study revealed that TET1 suppresses thyroid function by remodeling histone modifications in the *PAX8* promoter to downregulate the expression of itself and its downstream targets NIS and TPO.

## Materials and methods

### Transgenic mice

All animal studies were conducted with approval from the Laboratory Animal Center of Xi’an Jiaotong University (no. XJTU2019-G195). *LSL-Braf*^*V600E*^ mice were gifts from Prof. Mattin McMahon (The University of California). *TPO-Cre* mice were kindly provided by Prof. Shioko Kimura (National Cancer Institute). *Tet1*^*flox/flox*^ mice were purchased from Shanghai Model Organisms Center Inc. *LSL-Braf*^*V600E*^ mice and *TPO-Cre* mice were crossed to generate thyroid-specific *Braf*^*V600E*^ transgenic mouse model of thyroid cancer (*Thy-Braf*^*V600E*^) as previously described^15,16^. *Tet1*^*flox/flox*^ mice, *LSL-Braf*^*V600E*^ mice and *TPO-Cre* mice were crossed to generate thyroid-specific *Tet1* knockout combined with *Braf*^*V600E*^ transgenic mouse model of thyroid cancer (*Thy-Braf*^*V600E*^*; Tet1*^*−/−*^). Thyroid-specific *Tet1* knockout (*Thy-Tet1*^*−/−*^) mice were constructed by crossing *Tet1*^*flox/+*^ and *TPO-Cre* transgenic mice. Their wild-type (WT) littermates (*Thy-Braf*^*WT*^, *Thy-Braf*^*WT*^*; Tet1*^*+/+*^ or *Thy-Tet1*^*+/+*^) were used as the controls. These mice were kept in a specific pathogen-free environment.

All transgenic mice were weaned at 21 days of age, and genomic DNA was extracted from mouse tails for PCR analysis to determine the genotypes. The primer sequences are presented in Supplementary Table 1. Vital signs such as body weight, heart rate and blood pressure were measured at fixed time points during mouse breeding. Blood was collected from the orbital venous plexus of mice, and serum related indicators were then tested using ELISA kits according to the manufacturer’s protocol. The information of ELISA kits is presented in Supplementary Table 2. In addition, iodide uptake assays were conducted as previously described^17^.

### Dot-blot assay

Genomic DNA was isolated from mouse thyroid tissues by standard phenol-chloroform extraction. DNA was diluted in 2× SSC buffer to a concentration of 200 ng/μl and denatured with 0.1 M NaOH. Then 1,000 ng of genomic DNA was spotted onto a 0.45 μm positively charged nylon membrane (Roche Diagnostics), baked at 70°C for 5 min and fixed by UV crosslinking. The membrane was then washed, blocked with 5% casein buffer and incubated with an anti-5hmC antibody (Active Motif) overnight at 4 °C. After incubation with species-specific HRP-conjugated secondary antibody (ZSGB-BIO), the dot signal was visualized with an ECL detection kit (Millipore). Meanwhile, the same blot was stained with 0.02% methylene blue in 0.3 mol/l sodium acetate (pH 5.2) as a loading control.

### H&E staining

Mouse thyroid tissue sections were paraffin embedded, dewaxed, rehydrated and stained with hematoxylin and eosin (H&E) as previously described^18^. The images were then captured under an inverted microscope.

### TEM

Mice were sacrificed and thyroid and testicular tissues were dissected out. After rinsing with precooled saline, tissue blocks of less than 3 cubic millimeters in volume were rapidly cut. Next, the sections were postfixed with glutaraldehyde solution, dehydrated at room temperature, infiltrated and embedded, ultrathin sectioned and stained with uranyl acetate. The sections were observed with transmission electron microscopy (TEM) (Thermo Fisher).

### Western blotting analysis

The detailed protocol was described in a previous study^19^. Relative protein levels were quantified by ImageJ software. The antibodies used in this study are presented in Supplementary Table 3.

### Clinical samples

Under the ethics committee approval and patient consent, we collected 15 thyroid tissues from patients with hyperthyroidism and 15 normal thyroid tissues (distal noncancerous thyroid tissues) at the First Affiliated Hospital of Xi’an Jiaotong University and investigated TET1 expression by immunohistochemistry (IHC) staining. To measure serum exosomal miRNAs, the serum samples from 10 patients with hyperthyroidism and 10 healthy controls were obtained from the First Affiliated Hospital of Xi’an Jiaotong University.

### IHC staining

The IHC staining of the target proteins was performed as previously mentioned^17^. The percentage of positive cells were counted with ImageJ software. The antibodies used in this study are also described in Supplementary Table 3.

### RNA extraction and RT–qPCR

Total RNA from mouse thyroid tissues and cell lines were extracted by using Trizol. Quantitative PCR with reverse transcription (RT–qPCR) was performed according to previous studies^18^. Each assay was run in triplicate. *β-Actin* and *U6* were used as internal references to normalize the expression of the indicated genes and miR-29c-3p, respectively. The primer sequences used for RT–qPCR and miRNA reverse transcription are presented in Supplementary Tables 4–6.

### Cell culture

The human immortalized thyroid cell line HTori3 and thyroid cancer cell line 8505C were kindly provided by Prof. Haixia Guan (Guangdong Provincial People’s Hospital). The thyroid cancer cell line C643 was obtained from Dr. Lei Ye (Ruijin Hospital). All cell lines were routinely cultured in RPMI-1640 medium (Gibco) containing 10% fetal bovine serum (FBS).

### Transfection of shRNA

To establish TET1 stably knockdown cells, 1 × 10^5^ HTori3 and 8505C were cultured in 24-well plates and then transfected with lentivirus expressing short hairpin (sh)RNAs targeting TET1 (sh-TET1#1 and sh-TET1#2) or control lentivirus (sh-NC) when cell growth reached 40–50% confluence. Blasticidin was used for cell selection 2 days after transfection to achieve maximal knockout efficiency. All lentiviruses were obtained from Hanbio Biotechnology and the shRNA sequences are presented in Supplementary Table 7.

### Co-IP

For co-immunoprecipitation (co-IP), the cell lysates were incubated with primary antibodies or IgG overnight at 4 °C, followed by a 4-h incubation at 4 °C with protein A/G-agarose beads (Santa Cruz Biotechnology, sc2003). Immunoprecipitates were then washed with RIPA buffer and detected by western blotting analysis.

### HmeDIP–qPCR

The hdroxymethylated DNA immunoprecipitation-quantitative PCR (hmeDIP–qPCR) assay was conducted to assess the 5hmC levels of the *PAX8* promoter. Genomic DNA was extracted by phenol–chloroform and sonicated in fragments in 1× TE buffer. DNA was then denatured at 95 °C for 10 min and chilled on ice for 10 min. Ten percent of the DNA sample was extracted as an input reference. The remaining 90% of the sample was equally divided and incubated with 10 μl anti-5hmC and rabbit IgG overnight at 4 °C, followed by the addition of protein A/G agarose beads and incubation at 4 °C for 2 h. Next, antibody–chromatin complexes were washed three times with 1× IP buffer and digested with proteinase K at 48 °C for 3 h. The purified DNA was amplified for subsequent qPCR assays. The primer sequences are described in Supplementary Table 8.

### ChIP–qPCR

The enrichments of HDAC1, H3K9Ac and H3K27Ac on the promoter region of PAX8 were validated by chromatin immunoprecipitation-qPCR (ChIP-qPCR) following the ChIP kit instructions (9003, Cell Signaling Technology). In brief, HTori3 and 8505C cells were crosslinked with 37% formaldehyde and chromatin was fragmented by an ultrasonic cell disruptor. After immunoprecipitation with specific antibodies, the chromatin–protein complexes were eluted from 30 μl protein G magnetic beads and reversed crosslinked. The purified DNA fragments were then used for qPCR assays. All antibodies are presented in Supplementary Table 3 and the primer sequences are provided in Supplementary Table 9.

### Dual-luciferase reporter system

The pmirGLO-Vector plasmid was kindly provided by Prof. Yanke Chen (Xi’an Jiaotong University School of Health Science Center). TargetScan and miRbase were employed to predict the binding sites of miR-29c-3p in the 3′ untranslated region (3′ UTR) of *TET1*. Based on the predicted locations, PGLO-*TET1* *3*′*-UTR* WT and PGLO-*TET1* *3*′*-UTR* MUT plasmids were constructed by Tsingke Biotech. The above plasmids and miR-29c-3p mimics/mimics NC (RiboBio) were co-transfected into cells by using X-treme GENE HP transfection Reagent (Sigma). The luciferase intensity was then measured by the dual-luciferase reporter assay system (Promega) according to the manufacturer’s instructions. Luciferase activity was calculated based on the ratio of firefly luciferase activity versus Renilla luciferase activity.

### Extraction and identification of exosomes

Cell culture medium and serum were collected to extract exosomes by ultracentrifugation. The supernatant was centrifuged at 300*g* for 10 min, 2000*g* for 10 min and 120,000*g* for 70 min in sequence. Sterile PBS was used to resuspend the sediment of the exosomes. The exosomes were then collected and dripped onto a copper grid. The morphology of the exosomes was tested by TEM. The particle sizes of exosomes were analyzed by a particle size analyzer. Exosome-specific biomarkers CD9 and TSG101 were examined by western blot analysis.

### Statistical analysis

GraphPad Prism 8.3.0 software was employed to analyze all data. Data are shown as mean and s.d. The comparison between the two groups was analyzed by a Student’s *t* test, one-way analysis of variance (ANOVA) and two-way ANOVA. The SPSS statistical package (16.0) was used to calculate the statistical significance. Each experiment was performed in triplicate. *P* < 0.05 was considered statistically significant.

## Results

### Thyroid-specific *Tet1* knockout restores thyroid function of *Thy-Braf*^*V600E*^ mice

The thyroid-specific *Braf*^*V600E*^ transgenic mouse model (*Thy-Braf*^*V600E*^) was employed for studying orthotopic papillary thyroid carcinoma^20^. To generate this model, the *LSL-Braf*^*V600E*^ mice were crossed with *TPO-Cre* mice, where Cre recombinase expression was initiated in thyroid follicular epithelial cells at embryonic day 14.5. Genotypes of the offspring were identified by agarose gel electrophoresis of PCR products from mouse tails (Supplementary Fig. 1a). The thyroid glands of *Thy-Braf*^*V600E*^ mice were significantly bigger than those of WT mice (*Thy-Braf*^*WT*^) at 5 weeks after birth. H&E staining showed the thyroid glands were structurally disordered in *Thy-Braf*^*V600E*^ mice, characterized by less colloids in the follicular lumen, endomitotic process and pseudoinclusions (Supplementary Fig. 1b). In addition, body weights of *Thy-Braf*^*V600E*^ mice were obviously decreased compared with *Thy-Braf*^*WT*^ mice (Supplementary Fig. 1c). Compared with *Thy-Braf*^*WT*^ mice, *Thy-Braf*^*V600E*^ mice exhibited lower levels of serum free triiodothyronine (FT3), free tetraiodothyronine (FT4), total triiodothyronine (T3) and total tetraiodothyronine (T4), accompanied by elevated thyroid-stimulating hormone (TSH) levels over a follow-up period of 5 weeks (Supplementary Fig. 1d). These results, taken together, indicate that *Thy-Braf*^*V600E*^ mice develop severe hypothyroidism, which was consistent with a previous study^15^.

**Fig. 1:**
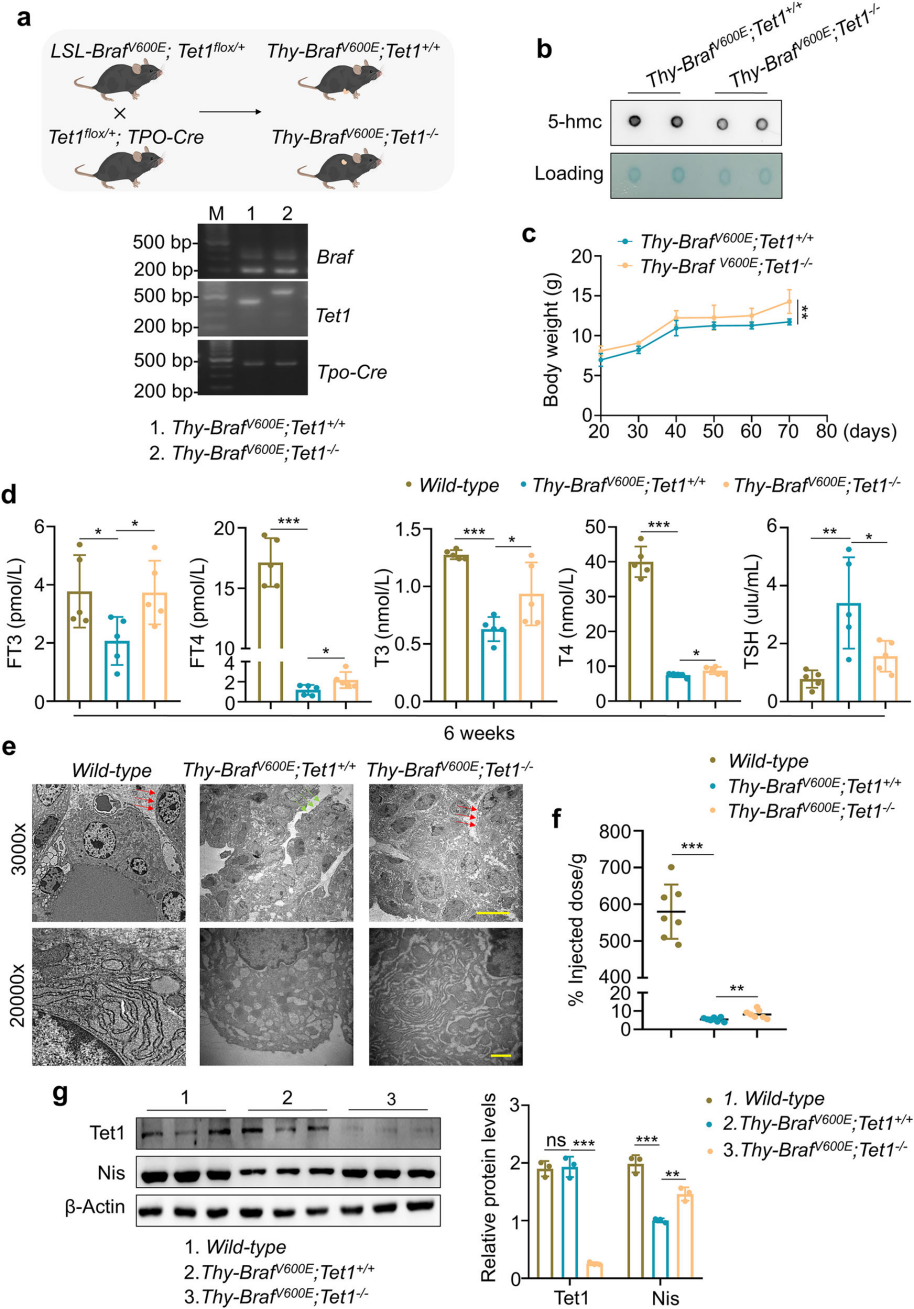
Thyroid-specific *Tet1* knockout restores thyroid function of *Thy-Braf*^*V600E*^ mice. **a** *LSL-Braf*^*V600E*^; *Tet1*^*flox/+*^ mice were crossed with *Tet1*^*flox/+*^; *TPO-Cre* mice to generate *Thy-Braf*^*V600E*^*; Tet1*^*−/−*^ (thyroid-specific *Tet1* knockout combined with *Braf*^*V600E*^ heterozygous mutation) and *Thy-Braf*^*V600E*^*; Tet1*^*+/+*^ (thyroid-specific *Braf* heterozygous mutation combined with WT *Tet1*) mice (top). Genotypes of these mice were identified by agar gel electrophoresis (bottom). M, 100 bp DNA Marker. **b** Dot-blot assays were performed to measure the 5hmC levels in thyroid tissues of *Thy-Braf*^*V600E*^*; Tet1*^*+/+*^ and *Thy-Braf*^*V600E*^*; Tet1*^*−/−*^ mice. **c** The changes in body weights of *Thy-Braf*^*V600E*^*; Tet1*^*+/+*^ and *Thy-Braf*^*V600E*^*; Tet1*^*−/−*^ mice from 3 to 10 weeks of age. **d** The serum levels of thyroid related hormones FT3, FT4, T3, T4 and TSH in 6-week-old WT (*Thy-Braf*^*WT*^*; Tet1*^*+/+*^), *Thy-Braf*^*V600E*^*; Tet1*^*+/+*^ and *Thy-Braf*^*V600E*^*; Tet1*^*−/−*^ mice were measured by chemiluminescence and ELISA (*n* = 5). **e** Representative TEM images of thyroid tissues from WT, *Thy-Braf*^*V600E*^*; Tet1*^*+/+*^ and *Thy-Braf*^*V600E*^*; Tet1*^*−/−*^ mice. The red arrows indicate abundant microvilli in the cell membrane and the green arrows indicate a very small amount of microvilli in the cell membrane of the lateral follicular cavity. Scale bars, 10 μm (3000×) and 1 μm (2000×). **f** Iodine uptake rates of thyroid gland in WT, *Thy-Braf*^*V600E*^*; Tet1*^*+/+*^ and *Thy-Braf*^*V600E*^*; Tet1*^*−/−*^ mice (*n* = 7). **g** The protein expression of Tet1 and Nis in thyroid tissues of WT, *Thy-Braf*^*V600E*^*; Tet1*^*+/+*^ and *Thy-Braf*^*V600E*^*; Tet1*^*−/−*^ mice was determined by western blotting analysis (left) and the quantitative results are shown (right). β-Actin was used as a loading control. The data are presented as the mean ± s.d. *P < 0.05, **P < 0.01, ***P < 0.001 and ns, not significant.

We previously generated a thyroid-specific *Tet1* knockout combined with thyroid-specific *Braf*^*V600E*^ transgenic mouse model (*Thy-Braf*^*V600E*^*; Tet1*^*−*^^*/−*^) and their control mice (*Thy-Braf*^*V600E*^*; Tet1*^*+/+*^) by crossing *LSL-Braf*^*V600E*^ mice, *Tet1*^*flox/+*^ mice and *TPO-Cre* mice to investigate the role of TET1 in thyroid cancer^17^. This model was constructed in the same way in the present study, and the genotypes of offspring are shown in Fig. 1a. Given the function of TET1 in converting 5mC to 5hmC, the 5hmC level can serve as an indicator of TET1 protein expression. As expected, dot-blot assays showed that 5hmC levels were substantially decreased in thyroid tissues of *Thy-Braf*^*V600E*^*; Tet1*^*−/−*^ mice compared with *Thy-Braf*^*V600E*^*; Tet1*^*+/+*^ mice (Fig. 1b), further indicating that TET1 was successfully knocked out in the thyroid glands of mice. Furthermore, we observed the changes in body weights of these mice from 3 weeks to 10 weeks after birth. The body weights of *Thy-Braf*^*V600E*^*; Tet1*^*−*^^*/−*^ mice were significantly increased compared with *Thy-Braf*^*V600E*^*; Tet1*^*+/+*^ mice (Fig. 1c). The growth retardation caused by *Braf*^*V600E*^ was somewhat improved.

The reproductive system serves as a critical target organ of thyroid hormones. Thus, normal levels of thyroid hormones are essential for the fertility of mice^21^. In fact*, Thy-Braf*^*V600E*^*; Tet1*^*+/+*^ mice were infertile due to severe impairment in thyroid function. Surprisingly, we found that *Thy-Braf*^*V600E*^*; Tet1*^*−/−*^ mice had restored reproductive ability, and significantly improved the rate of litter size compared with *Thy-Braf*^*V600E*^*; Tet1*^*+/+*^ mice (Table 1). Thus, we hypothesized that thyroid-specific *Tet1* knockout improved thyroid function in *Thy-Braf*^*V600E*^ mice. To prove this, we measured the levels of thyroid hormones in *Thy-Braf*^*V600E*^*; Tet1*^*−/−*^ mice, *Thy-Braf*^*V600E*^*; Tet1*^*+/+*^ mice and WT mice (*Thy-Braf*^*WT*^*; Tet1*^*+/+*^, as normal controls) by chemiluminescence and ELISA assays. As shown in Fig. 1d, the levels of FT3, FT4, T3 and T4 were significantly decreased in *Thy-Braf*^*V600E*^*; Tet1*^*+/+*^ mice compared with WT mice, accompanied by an obvious increase in TSH levels, while the above changes were partially reversed in *Thy-Braf*^*V600E*^*; Tet1*^*−/−*^ mice. We also observed the ultrastructural changes in the thyroid glands of these mice using TEM. As shown in Fig. 1e, compared with WT mice, the thyroid glands of *Thy-Braf*^*V600E*^*; Tet1*^*+/+*^ mice exhibited a loss of cell polarity of thyroid follicular epithelial cells and a lack of microvilli in the cell membrane of the lateral follicular cavity. Furthermore, the endoplasmic reticulum (ER) appeared swollen and expanded, suggesting abnormal protein synthesis. However, *Thy-Braf*^*V600E*^*; Tet1*^*−/−*^ mice had healthier thyroid structures than *Thy-Braf*^*V600E*^*; Tet1*^*+/+*^ mice, which was consistent with improved hormonal readiness. In addition, compared with WT mice, which usually have a strong ability to take up iodine, *Thy-Braf*^*V600E*^*; Tet1*^*+/+*^ mice exhibited an extremely weakened iodine uptake ability, whereas this effect was obviously alleviated in *Thy-Braf*^*V600E*^*; Tet1*^*−/−*^ mice (Fig. 1f). This was also supported by protein expression pattern of the sodium iodine transporter (NIS) in thyroid tissues of the above mice (Fig. 1g). These findings indicate that thyroid-specific *Tet1* knockout restores thyroid function of *Thy-Braf*^*V600E*^ mice.

**Table 1:** Pregnancy/mating and litter outcomes from pairing the indicated genotypes with C57BL/6J mice.

Genotypes	Pregnancy/mating %		Litter size (pup numbers)
	Male	Female	
*Thy-Braf*^*V600E*^*; Tet1*^*+/+*^	0 (*n* = 5)	–	0
*Thy-Braf*^*V600E*^*; Tet1*^*+/*^^*−*^	60 (*n* = 3)	–	3–13
*Thy-Braf*^*V600E*^*; Tet1*^*−/−*^	–	100 (*n* = 2)	6

### Thyroid-specific *Tet1* knockout restores the reproductive ability of *Thy-Braf*^*V600E*^ mice

**Fig. 2:**
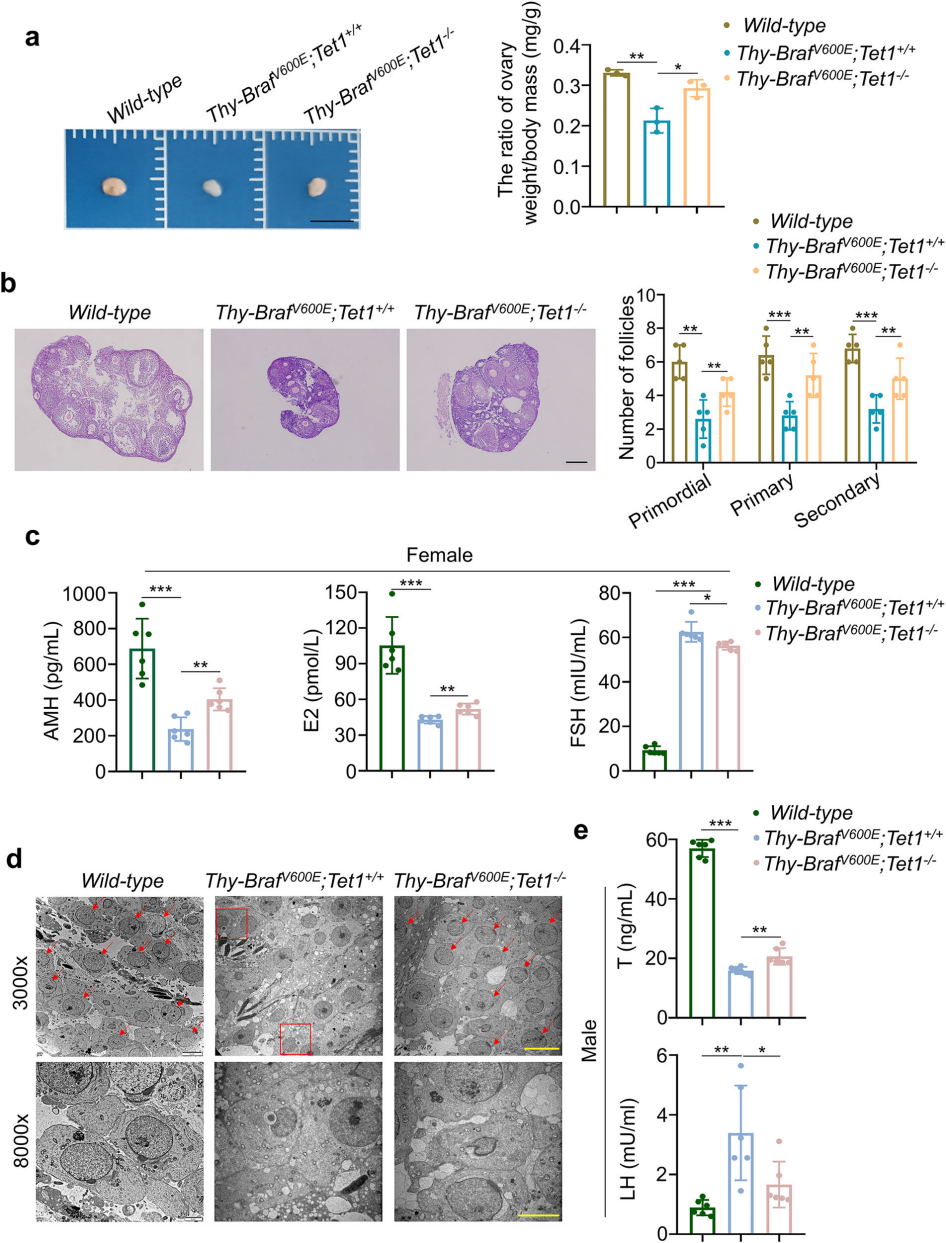
Thyroid-specific *Tet1* knockout restores the fertility of *Thy-Braf*^*V600E*^ mice. **a** Representative images of general ovaries (left) and the ratio of ovary weight to body mass (right) in 10-week-old female WT, *Thy-Braf*^*V600E*^*; Tet1*^*+/+*^ and *Thy-Braf*^*V600E*^*; Tet1*^*−/−*^ mice (*n* = 5). Scale bars, 0.5 cm. **b** Representative H&E staining of ovary tissues (left) and the numbers of primordial, primary and secondary follicles in 10-week-old female WT, *Thy-Braf*^*V600E*^*; Tet1*^*+/+*^ and *Thy-Braf*^*V600E*^*; Tet1*^*−/−*^ mice (right) (*n* = 5). Scale bars, 500 μm. **c** The serum levels of AMH, E2 and FSH in WT, *Thy-Braf*^*V600E*^*; Tet1*^*+/+*^ and *Thy-Braf*^*V600E*^*; Tet1*^*−/−*^ mice at 10 weeks of age (*n* = 6) were tested by ELISA. **d** Representative TEM images of testicle tissues in WT, *Thy-Braf*^*V600E*^*; Tet1*^*+/+*^ and *Thy-Braf*^*V600E*^*; Tet1*^*−/−*^ male mice. The red boxes indicate sperm cells with abnormal acrosomes, and red arrows indicate sperm cells with normal acrosomes. Scale bar,s 10 μm (3000×) and 1 μm (2000×). **e** The serum levels of testosterone (T) and luteinizing hormone (LH) in WT, *Thy-Braf*^*V600E*^*; Tet1*^*+/+*^ and *Thy-Braf*^*V600E*^*; Tet1*^*−/−*^ male mice (*n* = 6) were measured by ELISA. The data are presented as the mean ± s.d. *P < 0.05, **P < 0.01 and ***P < 0.001.

Our data have confirmed that thyroid-specific *Tet1* knockout can restore the fertility of *Thy-Braf*^*V600E*^ mice. To reinforce this conclusion, we observed the alterations in the reproductive system of these mice. As shown in Fig. 2a, the ovarian volumes of *Thy-Braf*^*V600E*^*; Tet1*^*+/+*^ mice were significantly smaller than WT mice, while *Thy-Braf*^*V600E*^*; Tet1*^*−/−*^ mice had larger ovarian volume and increased ovarian index (the ratio of ovarian weight to body weight) compared with *Thy-Braf*^*V600E*^*; Tet1*^*+/+*^ mice at 10 weeks of age. We also performed H&E staining to show different developmental stages of follicles in the ovarian. On the basis of their morphologic features, we observed decreased numbers of primordial, primary and secondary follicles in *Thy-Braf*^*V600E*^*; Tet1*^*+/+*^ mice compared with WT mice, and this effect could be effectively alleviated in *Thy-Braf*^*V600E*^*; Tet1*^*−/−*^ mice (Fig. 2b). Next, we measured the levels of reproductive hormones affected by ovarian and pituitary secretion in these mice by ELISA. Expectedly, *Thy-Braf*^*V600E*^*; Tet1*^*+/+*^ female mice had a decreased levels of anti-Mullerian hormone (AMH) and estradiol (E2), and an increased levels of follicle-stimulating hormone (FSH) compared with WT female mice at 10 weeks of age, whereas these effects could be partially reversed in *Thy-Braf*^*V600E*^*; Tet1*^*−/−*^ female mice (Fig. 2c). Ultrastructural analysis of testicular tissue by TEM showed vacuolated acrosome in sperm cells in testicular tissues of *Thy-Braf*^*V600E*^*; Tet1*^*+/+*^ male mice, but relatively normal in testicular tissues of *Thy-Braf*^*V600E*^*; Tet1*^*−/−*^ male mice, which were highly similar to WT mice (Fig. 2d). In addition, we examined the reproductive hormones levels in three groups of male mice. As shown in Fig. 2e, *Thy-Braf*^*V600E*^*; Tet1*^*+/*^^*−*^ male mice had lower serum levels of testosterone and higher levels of luteinizing hormone than WT male mice at 10 weeks of age, which were distinctly alleviated in *Thy-Braf*^*V600E*^*; Tet1*^*−/−*^ male mice. Collectively, these data demonstrate thyroid-specific *Tet1* knockout restores the reproductive ability of *Thy-Braf*^*V600E*^ mice.

### Thyroid-specific *Tet1* knockout mice manifest hyperthyroidism and a systemic hypermetabolic state

**Fig. 3:**
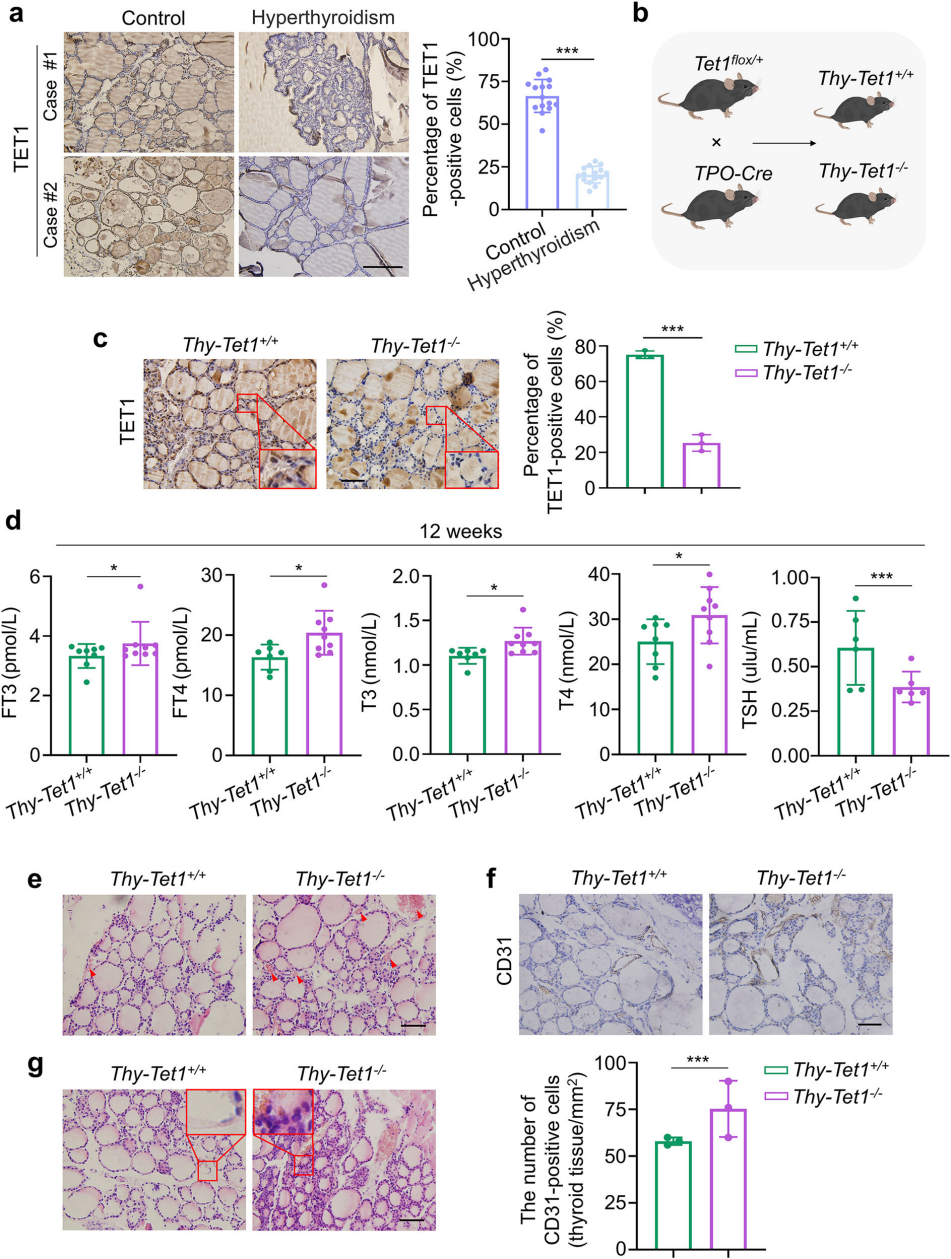
Thyroid-specific *Tet1* knockout mice exhibit a hyperthyroid phenotype. **a** Representative IHC staining of TET1 (left) and the statistical results of positive-stained cells (right) in thyroid tissues of patients with hyperthyroidism and healthy controls (*n* = 15). Scale bars, 200 μm. **b** *Tet1*^*flox/+*^ mice were crossed with *TPO-Cre* mice to generate thyroid-specific *Tet1* knockout (*Thy-Tet1*^*−/−*^) mice and WT (*Thy-Tet1*^*+/+*^) control mice. **c** Representative IHC staining of TET1 (left) and the statistical results of positive-stained cells (right) in thyroid tissues of *Thy-Tet1*^*+/+*^ and *Thy-Tet1*^*−/−*^ mice. Scale bars, 50 μm. **d** The serum levels of thyroid-related hormones FT3, FT4, T3, T4 and TSH in *Thy-Tet1*^*+/+*^ and *Thy-Tet1*^*−/−*^ mice (*n* = 6–9) were measured by chemiluminescence and ELISA. **e** H&E staining was used to observe the distribution of thyroid vessels in *Thy-Tet1*^*+/+*^ and *Thy-Tet1*^*−/−*^ mice. Scale bars, 50 μm. **f** Representative IHC staining of CD31 (top) and statistical results of positive-stained cells (bottom) in thyroid tissues of *Thy-Tet1*^*+/+*^ and *Thy-Tet1*^*−/−*^ mice. Scale bars, 50 μm. **g** Morphological observations of thyroid follicular epithelial cells in thyroid tissues of *Thy-Tet1*^*+/+*^ and *Thy-Tet1*^*−/−*^ mice by H&E staining. The data are presented as the mean ± s.d. *P < 0.05 and ***P < 0.001.

On basis of the above findings, we hypothesized that TET1 may negatively regulate thyroid function. To demonstrate this, we collected thyroid tissues from 15 patients with hyperthyroidism and 15 healthy individuals as controls at the First Affiliated Hospital of Xi’an Jiaotong University. IHC staining showed that the thyroid follicular epithelium exhibited papillary hyperplasia in thyroid tissues from patients with hyperthyroidism, accompanied by decreased expression of TET1 compared with the controls (Fig. 3a). To exclude the interference of *Braf*^*V600E*^, we crossed the *Tet1*^*flox/+*^ mice and *TPO-Cre* mice to generate thyroid-specific *Tet1* knockout (*Thy-Tet1*^*−/−*^) mice and their WT (*Thy-Tet1*^*+/+*^) control mice (Fig. 3b), and determined their genotypes by PCR analysis of tail DNA (Supplementary Fig. 2a). IHC staining showed that TET1 expression was significantly downregulated in the thyroid tissues of *Thy-Tet1*^*−/−*^ mice compared with *Thy-Tet1*^*+/+*^ mice (Fig. 3c). The levels of 5hmC in thyroid tissues of *Thy-Tet1*^*−/−*^ mice were consistently decreased compared with *Thy-Tet1*^*+/+*^ mice (Supplementary Fig. 2b). However, TET1 expression was not significantly different in the liver tissues of these two groups of mice (Supplementary Fig. 2c). These results confirmed that *Tet1* was specifically knocked out in thyroid glands of mice.

We next investigated the effect of thyroid-specific *Tet1* knockout on thyroid function of mice. As shown in Fig. 3d, the levels of FT3, FT4, T3 and T4 were significantly increased, while TSH levels were substantially decreased in *Thy-Tet1*^*−/−*^ mice compared with *Thy-Tet1*^*+/+*^ mice at the age of 12 weeks. Moreover, histopathological alterations of thyroid tissues were also different between these two groups of mice. Specifically, thyroid tissues of *Thy-Tet1*^*−/−*^ mice exhibited increased interfollicular vessels (Fig. 3e). CD31 is a platelet endothelial cell adhesion molecule that can be usually used to assess neovascularization^22^. Our data showed that the number of CD31-positive cells was obviously increased in thyroid tissues of *Thy-Tet1*^*−/−*^ mice relative to *Thy-Tet1*^*+/+*^ mice (Fig. 3f), which was consistent with the above results. In addition, H&E staining of thyroid tissues in *Thy-Tet1*^*−/−*^ mice showed that thyroid follicular epithelial cells exhibited a cubic and columnar morphology, accompanied by enlarged and darkly stained nuclei (Fig. 3g). These findings indicate that *Thy-Tet1*^*−/−*^ mice exhibit hyperthyroidism, which is consistent with the pathological findings of patients with hyperthyroidism.

In general, the immune indicators, such as TPOAb, TRAb, TGAb and TMAb, will be changed in the serum of patients with hyperthyroidism^23,24^. Thus, we investigated the effect of thyroid-specific *Tet1* knockout on these immune indicators in mice by ELISA. The results showed that the levels of TPOAb, TRAb, TGAb and TMAb did not differ significantly between *Thy-Tet1*^*+/+*^ and *Thy-Tet1*^*−/−*^ mice at 12 weeks of age (Supplementary Fig. 2d), suggesting that the levels of thyroid hormones in *Thy-Tet1*^*−/−*^ mice were not affected by immune-related factors but were autonomously synthesized and secreted by the glands. In addition, we examined thyroid glands of *Thy-Tet1*^*+/+*^ and *Thy-Tet1*^*−/−*^ mice using a small animal ultrasound imaging system, and failed to find a significant difference in the area of thyroid glands between these two groups of mice in the same cross-section (Supplementary Fig. 2e). Consistently, there was also no significant difference in thyroid weights between *Thy-Tet1*^*+/+*^ and *Thy-Tet1*^*−/−*^ mice (Supplementary Fig. 2f).

**Fig. 4:**
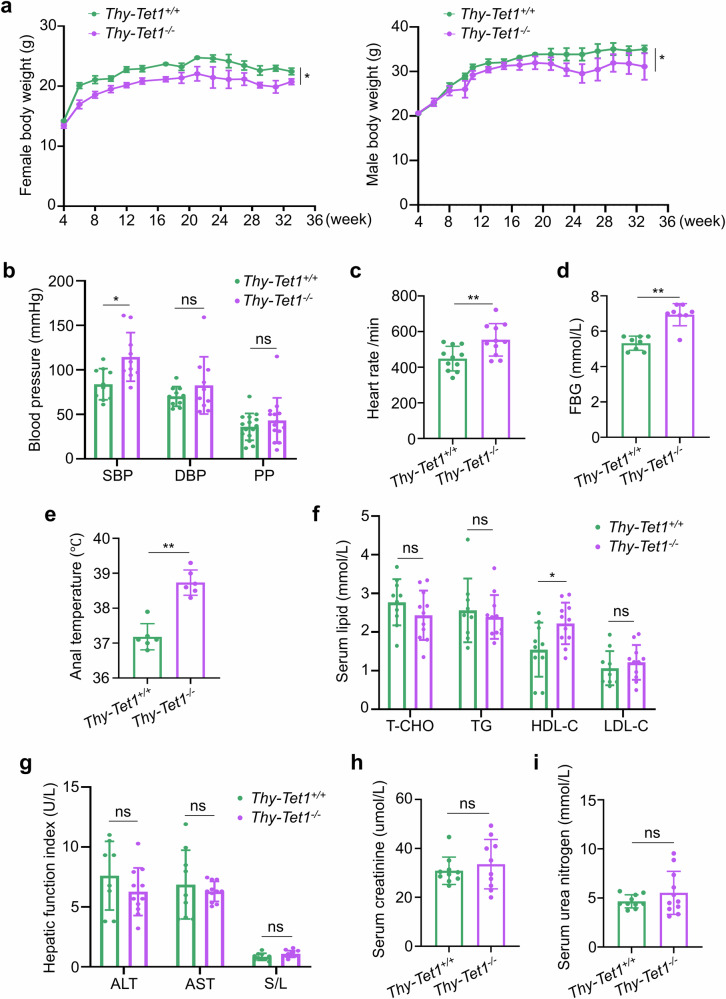
Thyroid-specific *Tet1* knockout mice present systemic hypermetabolic state. **a** The changes in body weights of female (left) and male (right) *Thy-Tet1*^*+/+*^ and *Thy-Tet1*^*−/−*^ mice from 4 to 36 weeks of age. **b**, **c** The SBP, diastolic blood pressure (DBP) and heart rate of *Thy-Tet1*^*+/+*^ and *Thy-Tet1*^*−/−*^ mice (*n* = 8–15) were detected by a tail-cuff method using the BP-2000 blood pressure analysis system. Pulse pressure (PP) = SBP − DBP. **d** The fasting blood glucose (FBG) of *Thy-Tet1*^*+/+*^ and *Thy-Tet1*^*−/−*^ mice (*n* = 8). **e** The anal temperature in *Thy-Tet1*^*+/+*^ and *Thy-Tet1*^*−/−*^ mice (*n* = 6). **f** The serum levels of total cholesterol (T-CHO), triglyceride (TG), HDL-C and low-density lipoprotein cholesterol (LDL-C) in *Thy-Tet1*^*+/+*^ and *Thy-Tet1*^*−/−*^ mice (*n* = 9–11) were measured by ELISA. **g** The serum levels of glutamic pyruvic transaminase (ALT) and glutamic oxalacetic transaminase (AST) were detected by ELISA, and AST/ALT ratios (S/L) were then calculated in *Thy-Tet1*^*+/+*^ and *Thy-Tet1*^*−/−*^ mice (*n* = 7–11). **h**, **i** The serum levels of creatinine (**h**) and urea nitrogen (**i**) in *Thy-Tet1*^*+/+*^ and *Thy-Tet1*^*−/−*^ mice (*n* = 10–11) were measured by ELISA. The data are presented as the mean ± s.d. *P < 0.05, **P < 0.01 and ns, not significant.

Patients with hyperthyroidism often have multiple symptoms owing to high levels of thyroid hormones^25^. We next evaluated several physiological measures of these two groups mice. From 4 to 36 weeks after birth, both female and male *Thy-Tet1*^*−/−*^ mice consistently exhibited lower body weights than *Thy-Tet1*^*+/+*^ mice (Fig. 4a). At 12 weeks, *Thy-Tet1*^*−/−*^ mice showed higher systolic blood pressure (SBP) (Fig. 4b), heart rate (Fig. 4c), fasting blood glucose (FBG) concentrations (Fig. 4d) and anal temperature (Fig. 4e) compared with *Thy-Tet1*^*+/+*^ mice. Analysis of blood lipid levels showed that *Thy-Tet1*^*−/−*^ mice had higher levels of high density lipoprotein cholesterol (HDL-C) than *Thy-Tet1*^*+/+*^ mice at 12 weeks (Fig. 4f). However, other indicators of liver and kidney function did not differ significantly between these two groups of mice during the observation period (Fig. 4g–i). These results suggest that *Thy-Tet1*^*−/−*^ mice suffer from hyperthyroidism-related systemic hypermetabolic symptoms.

### *Tet1* knockout causes a substantial upregulation of Pax8, Nis and Tpo

**Fig. 5:**
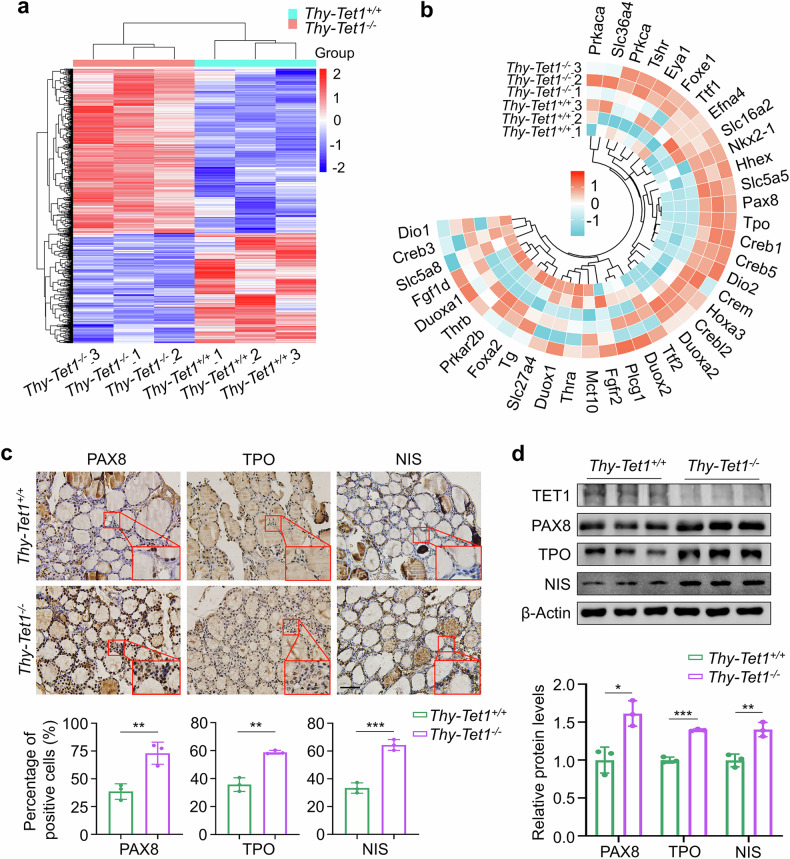
*Tet1* knockout upregulates Pax8, Nis and Tpo in thyroid tissues of mice. **a** A heat map of DEGs in the thyroid tissue of *Thy-Tet1*^*+/+*^ and *Thy-Tet1*^*−/−*^ mice. The log_2_ |FoldChange | > 0.585, *P* < 0.05. **b** A heat map of DEGs related to the synthesis and secretion of thyroid hormones. **c** Representative IHC staining of PAX8, TPO and NIS (top) and statistical results of positive-stained cells (bottom) in thyroid tissues of *Thy-Tet1*^*+/+*^ and *Thy-Tet1*^*−/−*^ mice. Scale bars, 50 μm. **d** The protein expression of TET1, PAX8, TPO and NIS in thyroid tissues of *Thy-Tet1*^*+/+*^ and *Thy-Tet1*^*−/−*^ mice was determined by western blotting analysis (top), and their quantitative results are shown (bottom). β-Actin was used as a loading control. The data are presented as the mean ± s.d. *P < 0.05, **P < 0.01, ***P < 0.001 and ns, not significant.

The aforementioned findings encourage us to investigate the mechanism by which TET1 negatively regulates thyroid function. To do this, we collected thyroid glands (*n* = 3 per group) from female *Thy-Tet1*^*+/+*^ and *Thy-Tet1*^*−/−*^ mice at 12 weeks and performed RNA sequencing (RNA-seq). Heat map analysis showed a total of 1,019 differentially expressed genes (DEGs) between thyroid tissues of these two groups of mice, of which 610 genes were upregulated and 409 genes were downregulated upon *Tet1* knockout (Fig. 5a). A volcano plot illustrates the distribution of genes with a log_2_|FoldChange | >0.585 significant difference (Supplementary Fig. 3a). We next performed Gene Ontology enrichment analysis, and found that the DEGs were mainly enriched in the regulation of hormone levels, hormone metabolic process, thyroid hormone metabolic process and so on(Supplementary Fig. 3b). Similarly, KEGG pathway analysis also revealed that the DEGs were enriched in thyroid hormone synthesis, thyroid hormone signaling pathway, PI3K–Akt signaling pathway and so on (Supplementary Fig. 3c).

The bioinformatics analysis strongly suggests that *Tet1* knockout may affect potential target genes and related signaling pathways, which are crucial for the synthesis and secretion of thyroid hormone. We then plotted a heat map of genes related to thyroid hormone synthesis and secretion to visualize these differences. The results showed that mRNA levels of *Pax8*, *Tpo* and *Slc5a5* (*Nis*) were obviously upregulated in thyroid tissues of *Thy-Tet1*^*−/−*^ mice compared with *Thy-Tet1*^*+/+*^ mice (Fig. 5b), as supported by the results of RT–qPCR (Supplementary Fig. 4). Consistently, IHC staining (Fig. 5c) and western blot analysis (Fig. 5d) showed a significant increase in protein expressions of PAX8, TPO and NIS in thyroid tissues of *Thy-Tet1*^*−/−*^ mice relative to *Thy-Tet1*^*+/+*^ mice. PAX8, a member of the paired box transcription factor family, acts synergistically with other transcription factors such as NKX2-1 (TTF1) and FOXE1 to regulate the development of thyroid follicular cells and the expression of thyroid-specific genes^26^. Previous studies have indicated that PAX8 is able to activate the transcription of *SLC5A5* and *TPO* by binding to their promoters^27,28^, and that NIS and TPO are required for synthesis and secretion of thyroid hormone^29^. Thus, we speculate that TET1 deletion may enhance thyroid function by promoting the transcription of *PAX8*.

### TET1 suppresses the transcription of *PAX8* by recruiting HDAC1 to its promoter

**Fig. 6:**
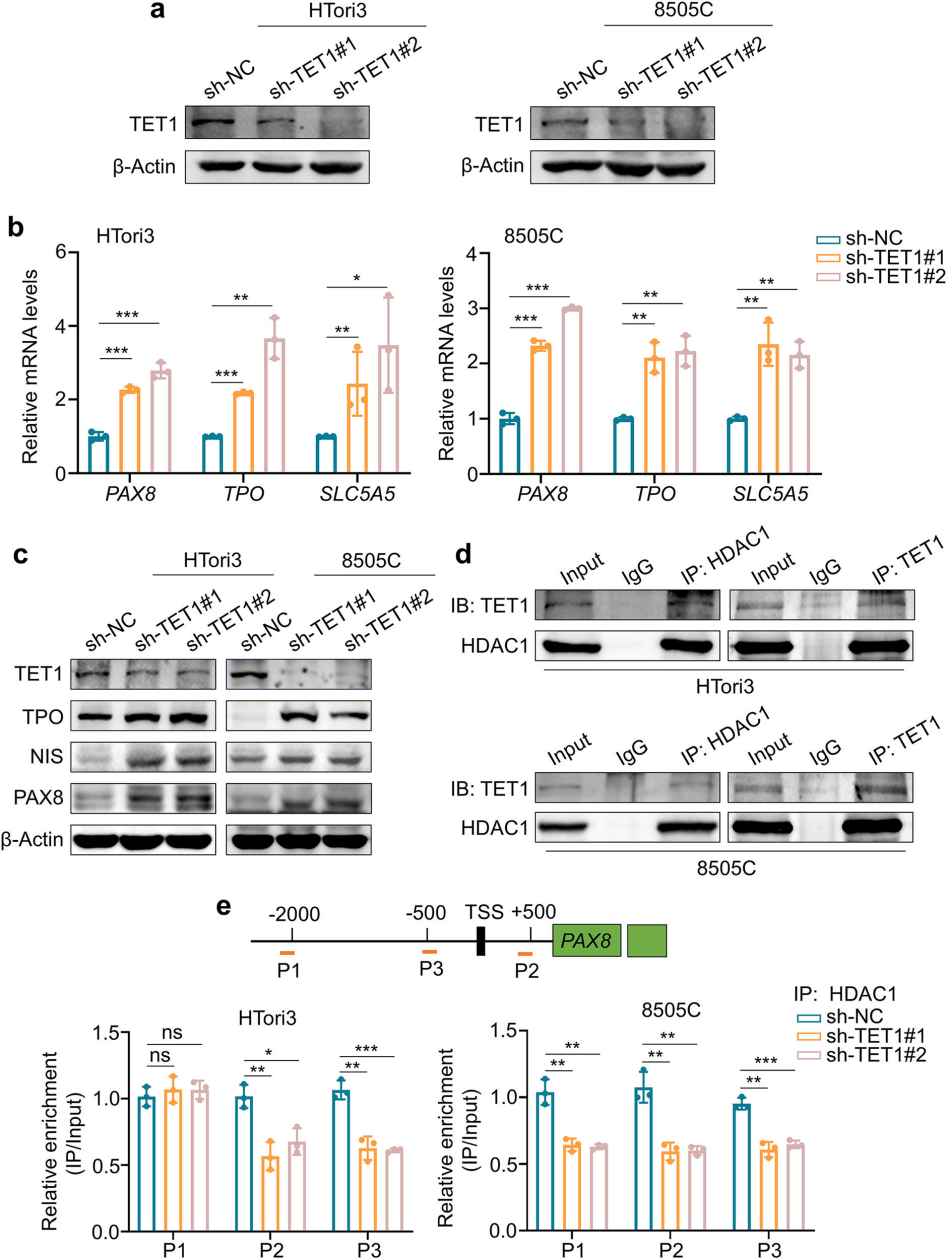
TET1 recruits HDAC1 to the promoter region of *PAX8.* **a** Validation of TET1 knockdown efficiency in HTori3 and 8505C cells by western blot analysis. β-Actin was used as a loading control. **b**, **c** RT–qPCR (**b**) and western blot (**c**) assays were used to determine mRNA and protein expression of PAX8, TPO and SLC5A5 in TET1-knockdown HTori3 and 8505C cells and their control cells. *β-Actin* was used as an internal reference gene for RT–qPCR, and β-Actin was used as a loading control for western blot. **d** Co-IP assays demonstrating the interaction between TET1 and HDAC1 in HTori3 and 8505C cells. **e** ChIP-qPCR assays were used to evaluate the binding of HDAC1 to the promoter of *PAX8* in TET1-knockdown HTori3 and 8505C cells and their control cells. P1, P2 and P3 represent three different amplifications in the promoter of *PAX8*. IgG antibody was used as a negative control. The data are presented as the mean ± s.d. *P < 0.05, **P < 0.01, ***P < 0.001 and ns, not significant.

To explore the mechanism by which TET1 regulates the transcription of *PAX8*, we stably knocked down TET1 in HTori3 and 8505C cells using a lentivirus system. Western blotting (Fig. 6a) and RT–qPCR (Supplementary Fig. 5) assays confirmed the knockdown efficiency of TET1 in these cells. Next, we examined the effect of TET1 knockdown on the expression of PAX8, NIS and TPO. The results showed that the mRNA (Fig. 6b) and protein (Fig. 6c) levels of PAX8, NIS and TPO were substantially upregulated in TET1-knockdown HTori3 and 8505C cells compared with control cells, which was consistent with the results in transgenic mice. As a DNA hydroxylase, TET1 could convert 5mC to 5hmC by binding to the promoters of target genes, thereby regulating their transcription. We demonstrated that the enrichment of 5hmC in the *PAX8* promoter was significantly decreased upon TET1 knockdown by hMeDIP–qPCR assays (Supplementary Fig. 6). However, reduced TET1 levels in the promoters of target genes usually inhibits their transcription^30^. Thus, we speculated that the TET1-mediated transcriptional repression of *PAX8* may involve mechanisms other than DNA demethylation.

TET1 has been shown to regulate gene expression by recruiting other factors to remodel chromatin structure via its nonenzymatic activity^8^. In addition, previous studies have demonstrated that TET1 interacts with histone deacetylase 1 (HDAC1) to regulate the transcription of target genes^31,32^, and there is report showing that transcription process of *PAX8* is regulated by HDAC1 in the development of normal embryonic kidney^31–33^. Thus, we hypothesized that TET1 may inhibit *PAX8* transcription by recruiting HDAC1 to its promoter region. To validate this, we first demonstrated a positive correlation between mRNA levels of *TET1* and *HDAC1* in normal thyroid tissues using the GEPIA database (Supplementary Fig. 7). Then, we predicted a potential interaction between TET1 and HDAC1 using the GeneMANIA database (Supplementary Fig. 8). This was supported by our co-IP assays in HTori3 and 8505C cells (Fig. 6d). Considering that HDAC1 is highly homologous to HDAC2, we thus analyzed the correlation between mRNA levels of *TET1* and *HDAC2* in normal thyroid tissues using the GEPIA database, and failed to find a significant association between them (Supplementary Fig. 9). We also performed a co-IP assay in HTori3 cells to further validate that there was no interaction between TET1 and HDAC2 (Supplementary Fig. 10). These observations indicate that TET1 represses the transcription of *PAX8* by recruiting HDAC1 rather than HDAC2 to reduce the acetylation levels within its promoter.

We next knocked down TET1 in HTori3 and 8505C cells and performed ChIP-qPCR assays in these cells using an anti-HDAC1 antibody. As shown in Fig. 6e, the enrichment of HDAC1 at two potential binding sites (P2 and P3) in the *PAX8* promoter was significantly reduced in TET1-knockdown HTori3 cells, while HDAC1 enrichment was consistently reduced at all three binding sites (P1–P3) in the *PAX8* promoter in TET1-knockdown 8505C cells compared with their control cells. In addition, we treated HTori3 and 8505C cells with different concentrations of the HDAC inhibitor vorinostat (SAHA). The results showed that SAHA upregulated the mRNA levels of *PAX8* in a concentration-dependent manner (Supplementary Fig. 11). These data, taken together, indicate that TET1 suppresses *PAX8* transcription by recruiting HDAC1 to its promoter.

### TET1 recruits HDAC1 to decrease the levels of H3K27Ac and H3K9Ac in the promoter of *PAX8*

**Fig. 7:**
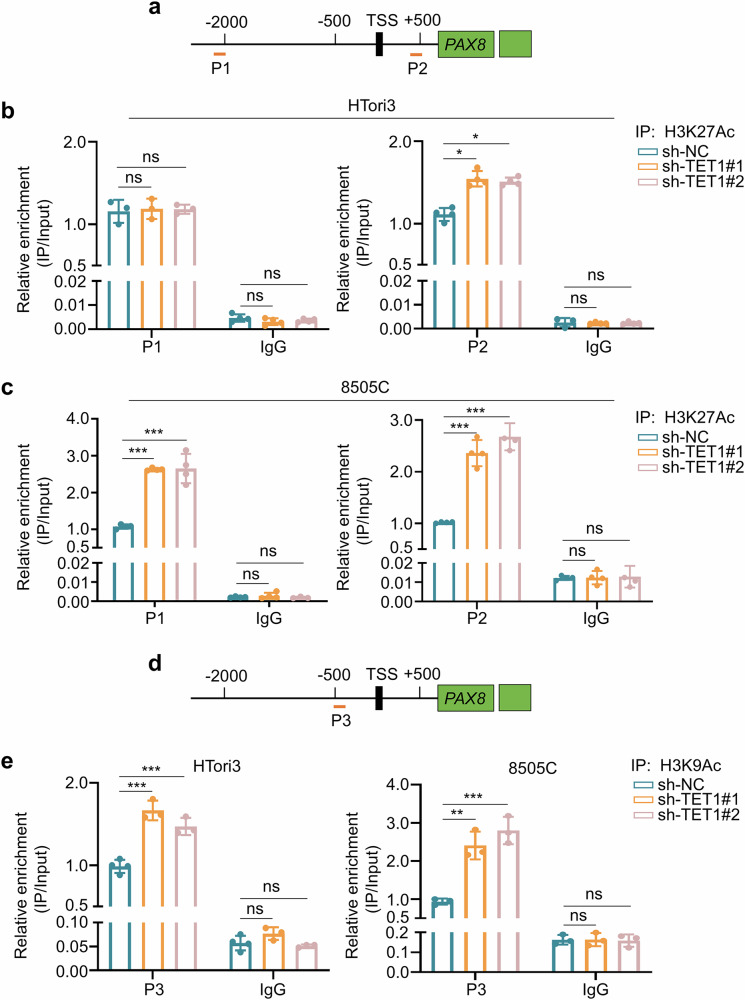
TET1 decreases the levels of H3K27Ac and H3K9Ac in the promoter of *PAX8.* **a** The primers were designed to amplify two regions (P1 and P2) of the promoter of *PAX8* to evaluate the levels of H3K27Ac. **b**, **c** ChIP-qPCR assays were used to determine the levels of H3K27Ac in the above two regions of *PAX8* promoter in TET1-knockdown HTori3 (**b**) and 8505C (**c**) cells and their control cells. **d** A pair of primers were designed to amplify one region (P3) of the promoter of *PAX8* to evaluate the levels of H3K9Ac. **e** The H3K9Ac levels in the above region of *PAX8* promoter were detected by ChIP-qPCR in TET1-knockdown HTori3 and 8505C cells and their control cells. IgG antibody was used as a negative control. The data are presented as the mean ± s.d. *P < 0.05, **P < 0.01, ***P < 0.001 and ns, not significant.

It has been reported that the H3K27 sites located within the P1 and P2 regions of *PAX8* promoter are prone to modification by HDAC1^34^, while the H3K9 site within the P3 region of its promoter was susceptible to modification by HDAC1^33^. Thus, we first designed specific primers to amplify the P1 and P2 regions (Fig. 7a). ChIP-qPCR assays demonstrated that TET1 knockdown significantly increased the enrichment levels of H3K27Ac in the P2 region but had no effect on the enrichment of H3K27Ac in the P1 region in HTori3 cells (Fig. 7b). However, H3K27Ac was markedly enriched in both P1 and P2 regions in TET1 knockdown 8505C cells (Fig. 7c). We also designed specific primers to amplify the P3 region (Fig. 7d), and proved that TET1 knockdown caused an increased enrichment of H3K9Ac in this region of the *PAX8* promoter in HTori3 and 8505C cells (Fig. 7e). These findings conclude that TET1 recruits HDAC1 to reduce the levels of H3K27Ac and H3K9Ac in the *PAX8* promoter. In addition, the transcription of *SLC5A5* has also been reported to be regulated by H3K9Ac in its promoter^35^. Thus, we designed two pairs of primers to amplify two regions (P1 and P2) in the *SLC5A5* promoter (Supplementary Fig. 12a) and demonstrated that TET1 knockdown did not affect the enrichment of H3K9Ac in these two regions of the *SLC5A5* promoter in HTori3 and 8505 C cells (Supplementary Fig. 12b).

**Fig. 8:**
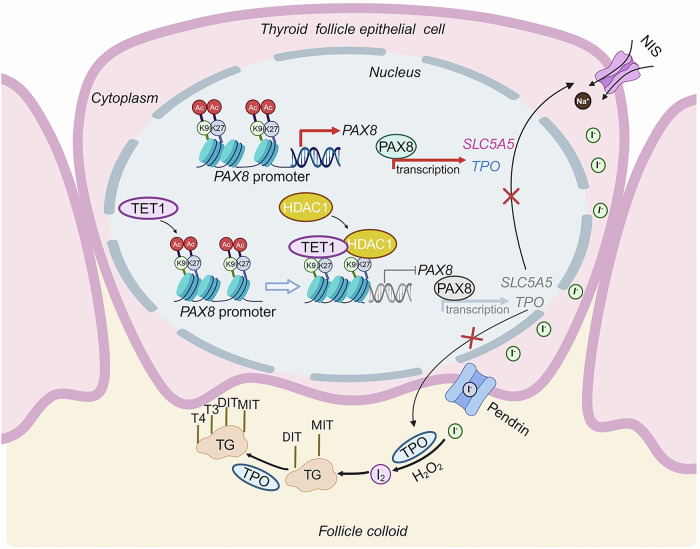
A schematic model for TET1 inhibiting thyroid function. TET1 inhibits the transcription of *PAX8* by recruiting HDAC1 to its promoter region to reduce the levels of H3K27Ac and H3K9Ac, which leads to the transcriptional repression of PAX8 downstream targets *SLC5A5* and *TPO* in thyroid follicular epithelial cells. Thus, decreased levels of TET1 may be a potential cause of hyperthyroidism. DIT diiodotyrosine, MIT monoiodotyrosine.

Taking the above results together, we have summarized the molecular mechanism how TET1 regulates thyroid function (Fig. 8). In brief, TET1 represses the transcription of *PAX8* by recruiting HDAC1 to reduce the levels of H3K27Ac and H3K9Ac in its promoter. As a result, the transcription of the PAX8 downstream targets *SLC5A5* and *TPO* is suppressed in thyroid follicular epithelial cells. Thus, TET1 inactivation may be a potential cause of hyperthyroidism.

### Elevated serum exosomal miR-29c-3p (exo-miR-29c-3p) potentially causes hyperthyroidism by targeting TET1

**Fig. 9:**
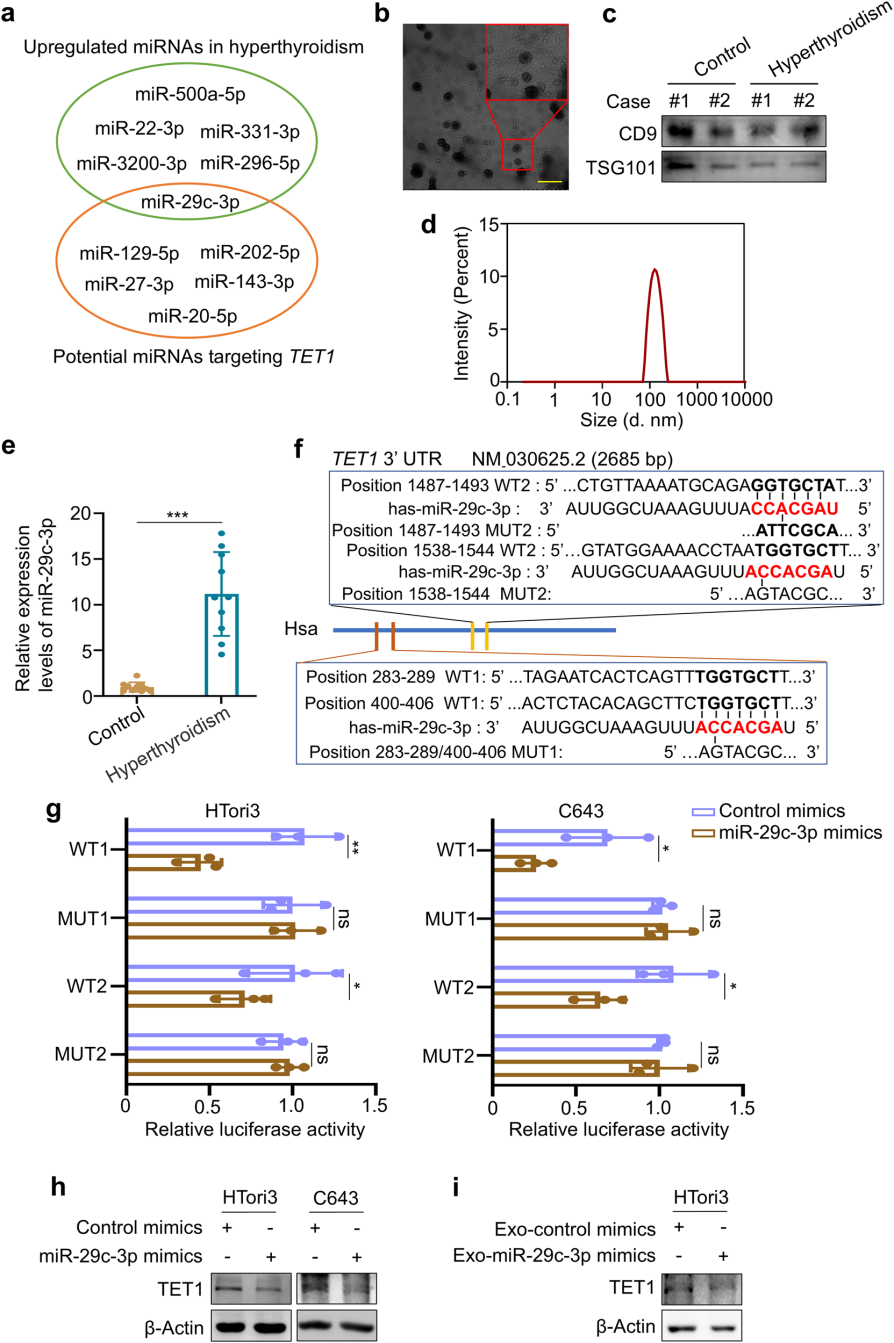
Exo-miR-29c-3p potentially regulates thyroid function by targeting TET1. **a** The intersection of elevated circulating miRNAs in the serum of patients with hyperthyroidism with miRNAs potentially targeting TET1. **b** The morphology of exosomes was observed by TEM. Scale bar, 200 nm. **c** Exosomes were isolated from the serum of patients with hyperthyroidism and healthy controls, and the surface proteins marker CD9 and TSG101 were then detected by western blot analysis. **d** The particle sizes of exosomes were detected by a particle size analyzer. **e** RT–qPCR was used to determine the levels of serum exosome miR-29c-3p (exo-miR-29c-3p) in patients with hyperthyroidism and healthy controls. *U6* was used as an internal reference gene. **f** Two WT and two mutant reporter plasmids were designed based on the sequences of predicated miR-29c-3p targeting *TET1 3*′*-UTR*. **g** miR-29c-3p mimics or control mimics and luciferase reporter plasmid pmirGLO containing WT (WT1 and WT2) and mutant (MUT1 and MUT2) were cotransfected into HTori3 and C643 cells. Dual-luciferase reporter assays were then performed to evaluate the binding of miR-29c-3p to the 3′ UTR of *TET*. **h** HTori3 and C643 cells were transfected with miR-29c-3p mimics or control mimics, and protein levels of TET1 were then detected by western blot analysis. β-Actin was used as a loading control. **i**, HTori3 cells were treated with exosomes isolated from the supernatants of miR-29c-3p mimics- and control mimics-transfected cells, and protein levels of TET1 were then determined by western blot analysis. β-Actin was used as a loading control. The data are presented as the mean ± s.d. *P < 0.05, **P < 0.01, ***P < 0.001 and ns, not significant.

A previous study indicated that the levels of circulating miRNAs were substantially elevated in patients with intractable Graves’ disease compared with healthy controls^36^. Moreover, the exosomes are the main form of intercellular communication, which regulate the function of target cells by transporting bioactive molecules including miRNAs^37,38^. Thus, we speculated that increased serum exosomal miRNAs may be a potential cause of hyperthyroidism. To validate this, we first intersected the upregulated miRNAs in the serum of patients with hyperthyroidism, with miRNAs potentially targeting TET1 that were predicted by miRbase and TargetScan online websites. Only one miRNA was obtained, which was miR-29c-3p (Fig. 9a). Next, we isolated the exosomes from the serum of patients with hyperthyroidism and healthy controls by ultracentrifugation and identified them using TEM (Fig. 9b). Exosome-specific biomarkers CD9 and TSG101 were also examined by western blot analysis (Fig. 9c). The average size of these exosomes measured by particle size analyzer was approximately 100 nm (Fig. 9d). These results indicated that the exosomes were successfully extracted. Serum exosomal miR-29c-3p (exo-miR-29c-3p) was then quantified by RT–qPCR assays, showing that the levels of exo-miR-29c-3p were significantly increased in the serum of patients with hyperthyroidism compared with healthy controls (Fig. 9e).

To verify the regulator effect of miR-29c-3p on TET1, we first transfected the miR-29c-3p mimics and control mimics into HTori3 and C643 cells, confirming transfection efficiency by RT–qPCR (Supplementary Fig. 13). Next, we constructed two WT reporter plasmids (WT1 and WT2) and two mutated reporter plasmids (MUT1 and MUT2) based on the sequences of predicated miR-29c-3p targeting *TET1 3*′*-UTR* (Fig. 9f). Dual-luciferase reporter assays showed that miR-29c-3p mimics attenuated the luciferase activity of WT plasmids, but not mutated plasmids in HTori3 and C643 cells compared with control mimics (Fig. 9g). Consistently, western blotting analysis further confirmed that TET1 expression was obviously reduced upon miR-29c-3p mimics in HTori3 and C643 cells compared with control mimics (Fig. 9h and Supplementary Fig. 14). Next, we transfected HTori3 cells with miR-29c-3p mimics and control mimics. After a 48-h transfection, we isolated the exosomes from the supernatants and named as exo-miR-29c-3p mimics and exo-control mimics. HTori3 cells were then treated with the above exosomes. The results showed that exo-miR-29c-3p mimics effectively decreased protein levels of TET1, as determined by western blot analysis (Fig. 9i and Supplementary Fig. 15). These findings, collectively, suggest that increased exo-miR-29c-3p levels may be a potential cause of hyperthyroidism by targeting TET1.

## Discussion

In *Thy-Braf*^*V600E*^ mice, key molecules involved in thyroid hormone synthesis such as SLC5A5 (NIS), TPO and TG are apparently downregulated, thereby leading to severe hypothyroidism. These mice thus lose fertility due to low levels of thyroid hormones^15,39,40^, also supported by our data. In addition, the present study unexpectedly discovered that thyroid-specific *Tet1* knockout could restore the fertility of *Thy-Braf*^*V600E*^ mice by improving their thyroid function. To further confirm this conclusion, we established a mouse model of thyroid-specific *Tet1* knockout (*Thy-Tet1*^*−/−*^). On the one hand, we aimed to exclude the interference of *Braf*^*V600E*^ mutation on thyroid function of mice. On the other hand, we attempted to determine the regulatory effect of Tet1 on thyroid function in mice. Our data showed that *Thy-Tet1*^*-/-*^ mice exhibited hyperthyroidism and a systemic hypermetabolic state, which was aligned with the symptoms of clinical hyperthyroidism.

The existing studies on TET1 mainly focus on its role in demethylation process that modulates malignant phenotypes and immune evasion of cancer cells^41–44^. However, the roles of TET1 in the functional maintenance of mature organs and the evolution of benign illness have rarely been studied. In recent years, the role of TET1 in metabolic diseases has received increasing attention. For example, it has been demonstrated that TET1 facilitates RXRα expression and adipogenesis via its DNA demethylation in 2021^45^. In the same year, a study indicated that *Tet1-*knockout mice developed abnormal glucose metabolism, resulting in impaired glucose homeostasis either fasting or after metformin injection. The specific mechanism is that TET1 deletion significantly downregulates the expression of *G6PC*, *PPAGGC1A* and *SLC2A4* related to hepatic glucose metabolism^46^. In addition, it has also been reported that TET1 deficiency in beige adipose led to increased energy expenditure in mice^31^. These observations suggest that, although TET1 plays a crucial role in hematologic malignancies and solid tumors, its importance in metabolic diseases should not be underestimated. Thus, the discovery that thyroid-specific *Tet1* knockout causes hyperthyroidism will be a meaningful extension for the role of TET1 in metabolic diseases.

The synthesis and secretion of thyroid hormones is mediated by the hypothalamic–pituitary–thyroid axis^47^. Specifically, TSH secreted from the anterior pituitary binds to its receptor TSHR on the surface of thyroid follicular cells and activates the downstream cAMP–PKA and PKC–IP3 signaling pathways. As a result, the expression of the vital transcription factors such as TTF1, TTF2, PAX8 and CREB are increased, which promote continuous synthesis of the essential functional proteins TPO, TG, NIS and DUOX1/2^48,49^. In the present study, compared with *Thy-Tet1*^*+/+*^ mice, *Thy-Tet1*^*−/−*^ mice developed hyperthyroidism, accompanied by elevated levels of PAX8, TPO and NIS. As a thyroid-specific transcription factor, PAX8 promotes the transcription of *TPO* and *SLC5A5* by binding to their promoters. In general, the regulation of TET1 on its downstream target genes depends on its demethylation activity. For example, TET1 facilitated the establishment of naive pluripotency by increasing 5hmC levels at somatic cell reprogramming target genes *Esrrb* and *Oct4*^50^. TET1 strengthened the demethylation of the *Sirt1* promoter and improved myocardial hypoxia/reoxygenation-mediated myocardial injury^51^. Moreover, TET1 also could maintain bone marrow mesenchymal stem cell homeostasis by the demethylation of the *P2rX7* promoter^52^. Normally, TET1 knockout will cause hypermethylation in the promoters of its downstream target genes, thereby repressing their expression. However, the present study showed that TET1 knockout upregulated PAX8 expression. This seems to contradict the above conclusion. Thus, we hypothesize that TET1 represses PAX8 expression independent of its demethylation activity. This was also supported by the results of our hMeDIP–qPCR assays.

It has been reported that TET1 knockdown has no significant effect on 5hmC content in the promoter regions of *PPARGC1A*, *G6PC* and *SLC2A4* involved in hepatic glucose metablism^46^. In addition, the nonenzymatic functions of TET1 have attracted increasing attention in recent years. For example, TET1 can repress the transcription of its target genes by recruiting histone modification-related complexes such as ARID4B/SIN3A and polycomb group protein EZH2 to their promoter regions^53,54^. Conversely, TET1 interacts with histone acetylase MOF to increase the levels of H4K16 acetylation in the promoter regions of its target genes, thereby activating their transcription^55^. In the present study, we demonstrated that TET1 inhibits *PAX8* transcription by interacting with HDAC1 to reduce the levels of H3K27Ac and H3K9Ac in its promoter. Similarly, a previous study showed that TET1 recruited HDAC1 to the promoters of thermogenesis-related genes *UCP1* and *PPARGCLA* in beige adipose tissue, thereby attenuating their transcription^31^. In addition, the enzymatic activity of HDAC1 was demonstrated to be crucial for the expression of *PAX2/PAX8*, *GDNF*, *SFRP1* and *CDKN1A* in embryonic kidney cells and the process of cell growth and differentiation. The HDAC1 inhibitor substantially promoted the transcription of the above genes by increasing the acetylation levels in their promoters^33^.

To be consistent with the results of the above animal studies, we demonstrated that TET1 expression was significantly downregulated in thyroid tissues of patients with hyperthyroidism compared with healthy controls. However, there are many reasons for its downregulation. Among them, the exosomes as the main form of intercellular communication regulate gene expression through transporting miRNAs to target cells^38^. The present study showed that the levels of miR-29c-3p were evidently elevated in the serums of hyperthyroid patients compared with healthy controls, and demonstrated that exo-miR-29c-3p inhibited the expression of TET1 in target cells. In support, a previous study indicated that miR-29c negatively regulated TET1 in different types of cancers^56,57^. In addition, our previous study observed a similar phenomenon showing that the levels of exo-miR-146a-5p was significantly upregulated in subclinical hypothyroidism and elevated exo-miR-146a-5p suppressed thyroid function by targeting the NG2/c-Myc/TSHR signaling axis^58^. These observations, taken together, suggest that elevated exo-miR-29c-3p may be one of the potential causes of hyperthyroidism.

In summary, we have established a transgenic mouse model with thyroid-specific *Tet1* knockout and found that TET1 could inhibit the synthesis and release of thyroid hormones. Furthermore, we elucidated the molecular mechanism by which TET1 suppressed thyroid function, and confirmed that elevated miR-29c-3p in serum exosomes was involved in regulating thyroid function and disease progression via targeted inhibition of TET1. Although the present study has some limitations, such as a limited number of clinical samples and the unknown specific binding sites between TET1 and HDAC1, it expands our understanding of the biological function of TET1 in metabolic diseases and provides a new perspective to reveal the pathogenesis of hyperthyroidism.

## Supplementary information

Supplementary Information

## Data Availability

RNA-seq data are available in the NCBI Gene Expression Omnibus (GSE281167).
