# Supplementary material for: TET1 loss propels the development of hyperthyroidism by remodeling histone modifications of PAX8 promoter
Source: Exp Mol Med. 2025 Oct 29;57(10):2376–92. doi: 10.1038/s12276-025-01566-2 (PMC12586522; doi:10.1038/s12276-025-01566-2)
Supplement: Supplementary file 1 — Supplementary Information [file 12276_2025_1566_MOESM1_ESM.pdf]

Supplementary Data

Supplementary Table 1. The primers used in this study for genotyping

**Genes**	**Sequence (5’-3’)**
Mouse-*Braf* (Sense)	TGAGTATTTTTGTGGCAACTGC
Mouse-*Braf* (Antisense)	CTCTGCTGGGAAAGCGGC
Mouse-*Tet1* (Sense)	CATCCTAAATAACCCAACCACCAA
Mouse-*Tet1* (Antisense)	ACAGTAGTATTTTGCCTGCCTGCAT
Mouse-*Tpo* (Sense)	AGGTGTAGAGAAGGCACTTAGC
Mouse-*Tpo* (Antisense)	CTAATCGCCATCTTCCAGCAGG

Supplementary Table 2. The ELISA kits used in this study

**Items**	**Source**
T3/T4/FT3/FT4	Roche
TSH	CUSABIO
T	FANKEW
LH	mlbio
AMH	mlbio
FSH	FANKEW
E2	FANKEW
TPOAb	mlbio
TRAb	mlbio
TGAb	mlbio
TMAb	mlbio
T-CHO	Nanjing Jiancheng
TG	Nanjing Jiancheng
HDL-C	Nanjing Jiancheng
LDL-C	Nanjing Jiancheng
ALT	Nanjing Jiancheng
AST	Nanjing Jiancheng
Cr	Nanjing Jiancheng
BUN	Nanjing Jiancheng

Supplementary Table 3. The antibodies used in this study

**Antibodies**	**Source**	**Assays**
5hmC	Active Motif	WB/IHC/Dot-blot
NIS	Proteintech	WB/IHC
β-ACTIN	Abways	WB
PAX8	Proteintech	WB/IHC
TSHR	Santa Cruz	WB/IHC
TG	Zenbio	WB/IHC
TET1	GeneTex	IP/IHC/WB
TPO	Santa Cruz	WB/IHC
HDAC1	Invitrogen	IP/WB/ChIP
H3K27Ac	Abcam	ChIP
H3K9Ac	Abmart	ChIP
CD63	Santa Cruz	WB
TSG101	Santa Cruz	WB
HDAC2	Proteintech	IP/WB/ChIP

Supplementary Table 4. The primers used in this study for qRT-PCR

**Genes**	**Sequence (5’-3’)**
Mouse-*Pax8* (Sense)	ATTCACAAAGGCCCCTCCTA
Mouse-*Pax8* (Antisense)	CGTAGGAAAGCTGCGAGTGT
Mouse-*Slc5a5* (Sense)	AGTACCTAGAACTGCGCTTCA
Mouse- *Slc5a5* (Antisense)	CAGGGTCAAAGTCCATCAGGT
Mouse-*Tpo* (Sense)	TGTCCAGTGTCTTGAGCTG
Mouse-*Tpo* (Antisense)	TGCCGATCACAGAGAGAGTG
Mouse-*Tshr* (Sense)	CTCGAAAATGCAAGACTGGG
Mouse-*Tshr* (Antisense)	CCAGGAGGACGACTTCAGAG
Mouse-*Tg* (Sense)	GCTGAGACAGGACTGGAATTGT
Mouse-*Tg* (Antisense)	CGGACTGAAGTAGTCTGGGG
Mouse-*β-Actin* (Sense)	ATGGAGGGGAATACAGCCC
Mouse-*β-Actin* (Antisense)	TTCTTTGCAGCTCCTTCGTT
Human-*PAX8* (Sense)	CAGGTCTACGATGCGCTG
Human-*PAX8* (Antisense)	TGCCTCACAACTCCATCAGA

Human-*SLC5A5* (Sense)	CTCCCTGCTAACGACTCCAG
Human-*SLC5A5* (Antisense)	GAGGTCCCACCACAACAATC
Human-*TPO* (Sense)	CGGGTCATCTGTGACAACAC
Human-*TPO* (Antisense)	CGGAGTCTACGCAGGTTCTC
Human-*TSHR* (Sense)	CTTGCTGGACGTGTCTCAAA
Human-*TSHR* (Antisense)	CTGGCCAAAACCAATGATCT
Human-*TG* (Sense)	AGGCCCTGCTCTCTAACTCC
Human-*TG* (Antisense)	GCCAAAGGAGTGCTGAAGTC
Human-*TET1* (Sense)	GCCAACCTTAGGGAGTAACAC
Human-*TET1* (Antisense)	TTGCGTCATTCTTCAGTGGA
Human-*β-ACTIN* (Sense)	CCTTGCACATGCCGGAG
Human-*β-ACTIN* (Antisense)	GCACAGAGCCTCGCCTT

Supplementary Table 5. The primers used in this study for miRNA reverse transcription

**Genes**	**Sequence (5'-3')**
miR-29c-3p	GTCGTATCCAGTGCGTGTCGTGGAGTCGGCAATTGCA CTGGATACGACTAA
U6	CGCTTCACGAATTTGCGTGTCAT

Supplementary Table 6. The primers used in this study for miRNA qRT-PCR

**Genes**	**Sequence (5'-3')**
miR-29c-3p (Sence)	TGCTTAGCACCATTTGAAA
miR-29c-3p (Antisence)	ATCCAGTGCGTGTCGTG
U6 (Sence)	GCTTCGGCAGCACATATACTAAAAT
U6 (Antisence)	CGCTTCACGAATTTGCGTGTCAT

Supplementary Table 7. shRNAs used in this study

shRNAs	Sequence (5'-3')
sh-NC	GATCCGTTCTCCGAACGTGTCACGTAATTCAAGAGAT TACGTGACACGTTCGGAGAATTTTTTC
sh-TET1#1	ACACAACTTGCTTCGATAATT
sh-TET1#2	CCACTTTCTAAGGGTTTAGA

Supplementary Table 8. The primers used in this study for hMeDIP-PCR

Fragments	Sense (5'-3')	Antisense (5'-3')
PAX8-CpG#1	GCCTCAAGGTGGGCCGAGGG GT	CGGGGAGGGAACACGCACAAGC CAA
PAX8-CpG#2	CCTCTTCCCTCCGCT	CATCCACCCGGCATC

Supplementary Table 9. The primers used in this study for ChIP -qPCR

Fragments	Sense（5'-3'）	Antisense（5'-3'）
PAX8-P1	AGAAGAGGTACTGTCAAG	ATCCAGCAAGTTATCAGA
PAX8-P2	AATCTCATCGCATCTCAT	GATCTGATGGAGTTGTGA
PAX8-P3	CAGAGGGAATGGCTTCAGGT	TCTGTAGGATGCCCTCCTTG
SLC5A5-P1	GAGTGCTGAAGCAGGCTGTGC	GGGAGCAGCTCGTGATTGTGG
SLC5A5-P2	CTGGCACAGGGCCAACTCTCA	TCAGGGTTTCAGGGGACCCATA

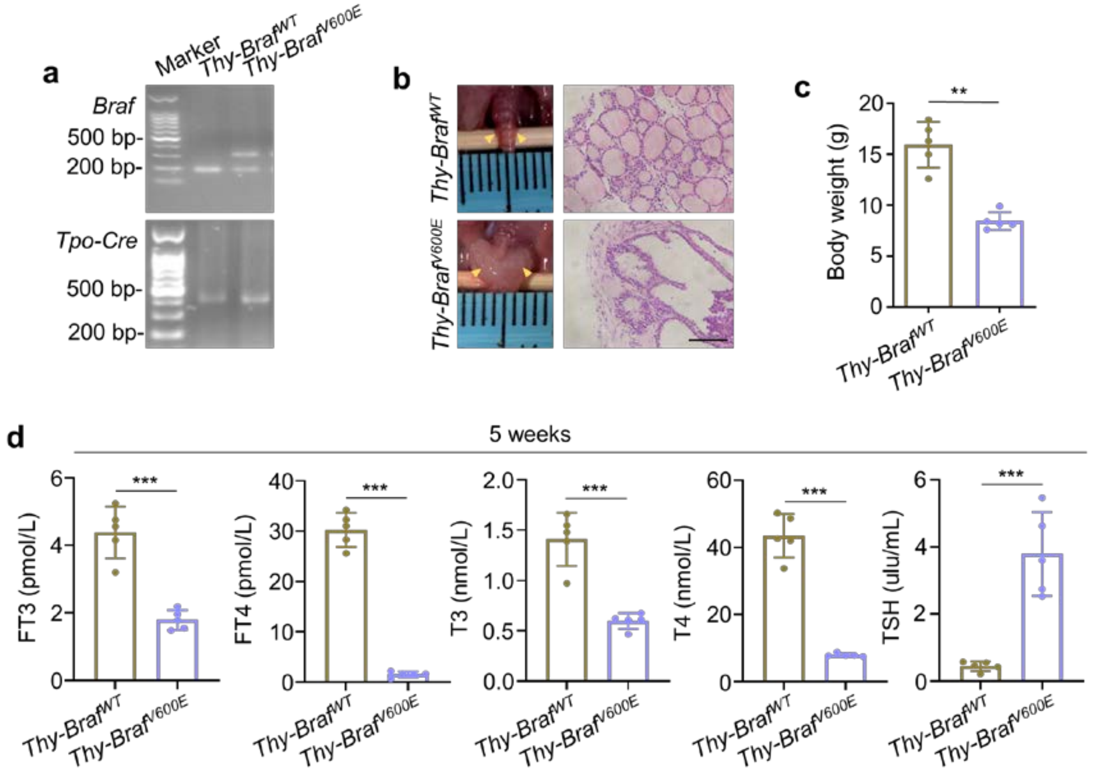

Supplementary Fig. 1 *Thy-Braf*^{V600E} mice develop severe hypothyroidism. a The genotypes of *Thy-Braf*^{V600E} (thyroid-specific *Braf*^{V600E} heterozygous mutation) mice and *Thy-Braf*^{WT} (*Braf* wild-type) were identified by agar-gel electrophoresis. Marker, 100 bp DNA Marker. **b** Representative images of thyroid tumors (left panels) and H&E staining (right panels) of thyroid tissues in 5-week-old *Thy-Braf*^{WT} and *Thy-Braf*^{V600E} mice. Yellow triangles indicate thyroid glands. Scale bar, 100 μm. **c** The body weights of 5-week-old *Thy-Braf*^{WT} and *Thy-Braf*^{V600E} mice. **d** The serum levels of thyroid related hormones FT3, FT4, T3, T4 and TSH in *Thy-Braf*^{WT} and *Thy-Braf*^{V600E} mice. The data were presented as the mean ± SD. **, $P < 0.01$; ***, $P < 0.001$.

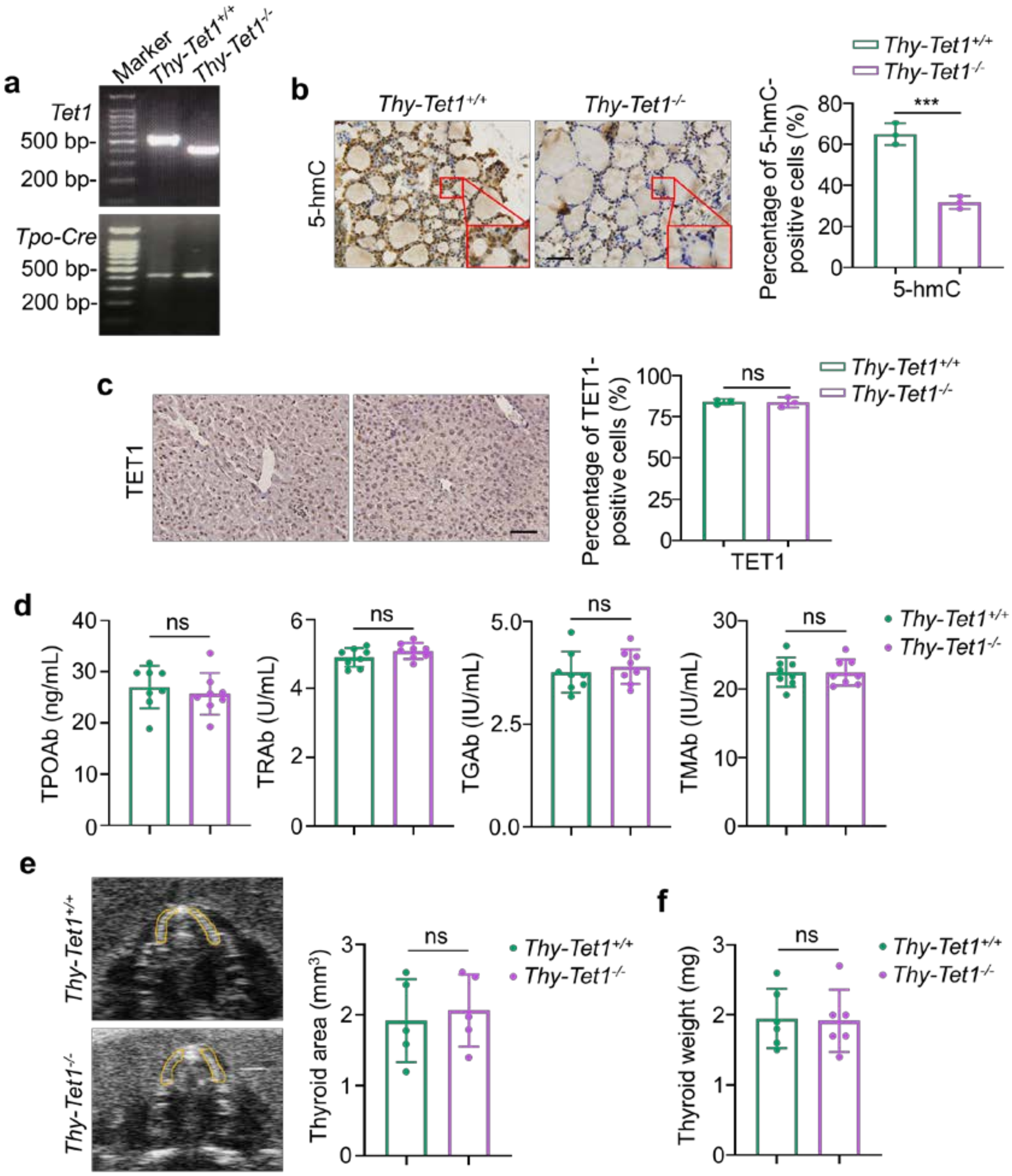

Supplementary Fig. 2 a The genotypes of *Thy-Tet1*$^{+/+}$ and *Thy-Tet1*$^{-/-}$ mice were identified by agar-gel electrophoresis. Marker, 100 bp DNA Marker. **b** Representative IHC staining of 5-hmC in thyroid tissues of *Thy-Tet1*$^{+/+}$ and *Thy-Tet1*$^{-/-}$ mice (left panels) and their statistical results of positive-stained cells (right panel). Scale bar, 50 μm. **c** Representative IHC staining of TET1 in liver tissues of *Thy-Tet1*$^{+/+}$ and *Thy-Tet1*$^{-/-}$ mice (left panels) and

their statistical results of positive-stained cells (right panel). Scale bar, 50 μm. **d** The serum levels of thyroid peroxidase antibody (TPOAb), TSH receptor antibody (TRAb), thyroglobulin antibody (TGAb) and thyroid microsomal antibody (TMAb) in *Thy-Tet1*$^{+/+}$ and *Thy-Tet1*$^{-/-}$ mice (n =7-8) were measured by ELISA. **e** The areas of thyroid glands between *Thy-Tet1*$^{+/+}$ and *Thy-Tet1*$^{-/-}$ mice (n = 5) were measured by a small animal ultrasound imaging system (left panels) and their statistical results were shown in the right panel. **f** The thyroid weights of *Thy-Tet1*$^{+/+}$ and *Thy-Tet1*$^{-/-}$ mice (n = 6). The data were presented as the mean ± SD. ***, $P < 0.001$; ns, no significance.

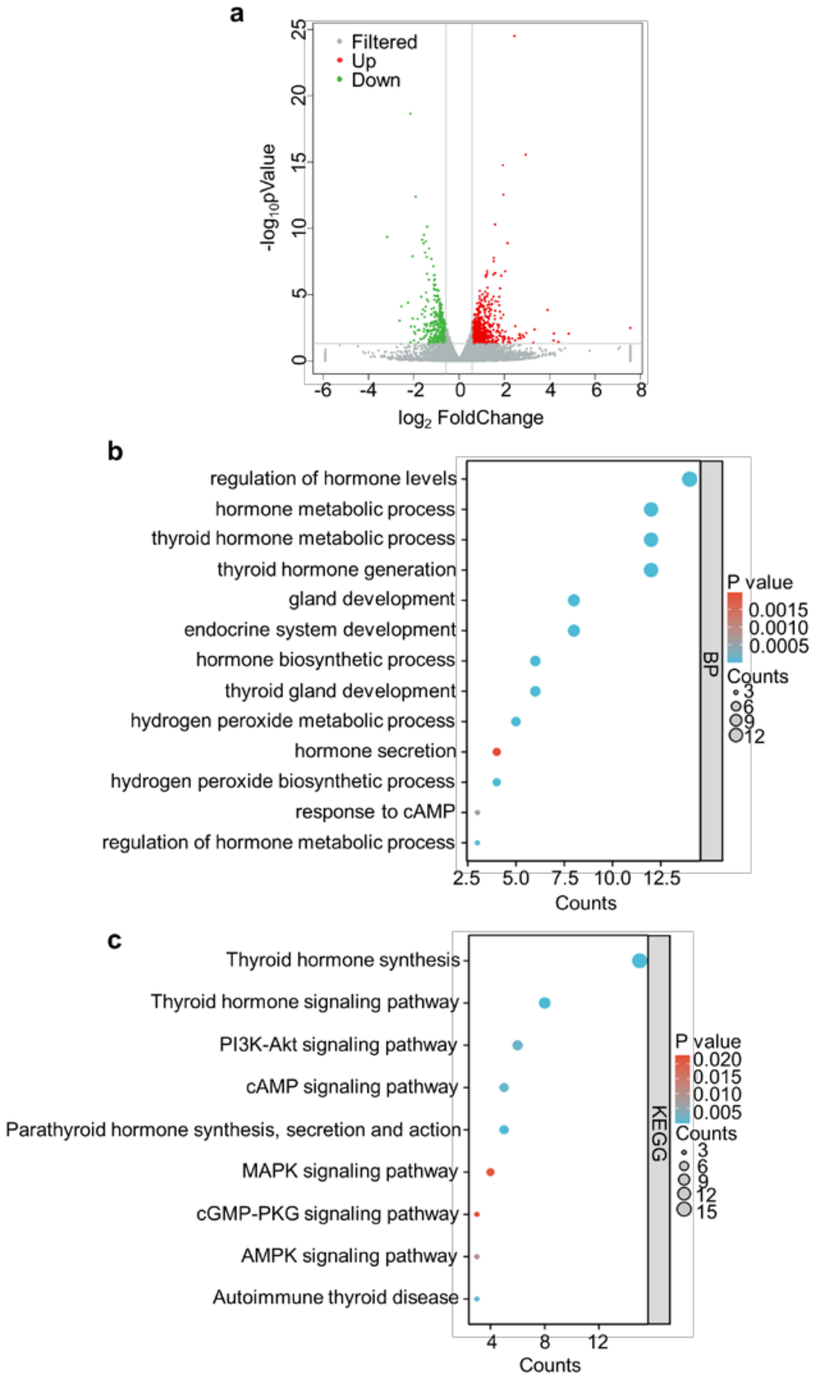

Supplementary Fig. 3 a The volcano map for the visualization of differentially expressed genes in thyroid tissues of *Thy-Tet1*$^{+/+}$ and *Thy-Tet1*$^{-/-}$ mice. Log2|FoldChange| > 0.585, P < 0.05. GO (**b**) and KEGG (**c**) analyses of differentially expressed genes in thyroid tissues of *Thy-Tet1*$^{+/+}$ and *Thy-Tet1*$^{-/-}$ mice (n = 6).

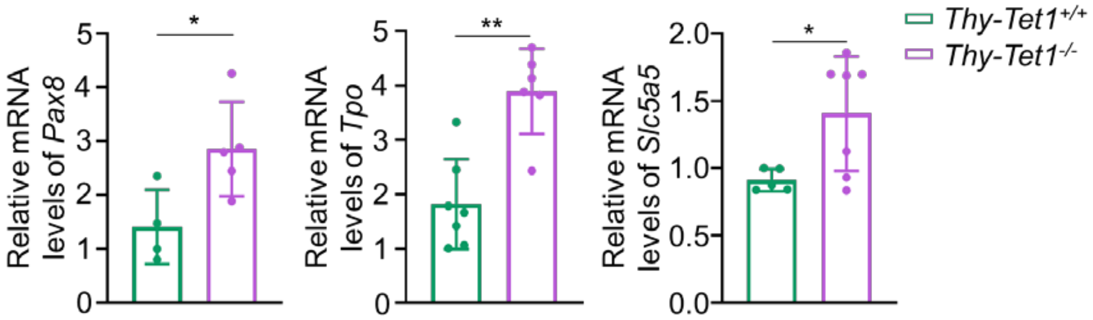

Supplementary Fig. 4 mRNA levels of *Pax8*, *Tpo* and *Slc5a5* in thyroid tissues of *Thy-Tet1*$^{+/+}$ and *Thy-Tet1*$^{-/-}$ mice were determined by qRT-PCR. *β-Actin* was used as an internal reference gene. The data were presented as the mean ± SD. *, *P* < 0.05; **, *P* < 0.01.

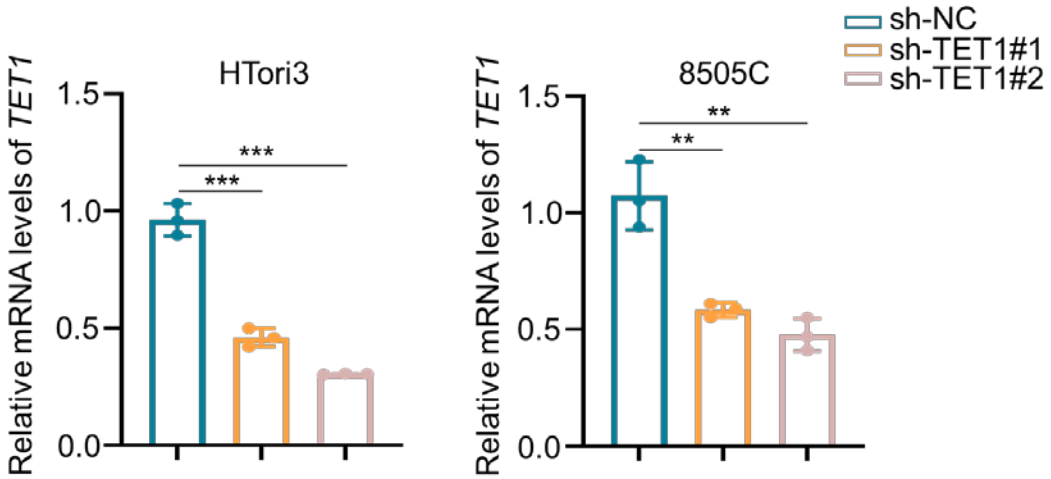

Supplementary Fig. 5 The knockdown efficiency of TET1 was determined by qRT-PCR. *β-Actin* was used as an internal reference. The data were presented as the mean ± SD. **, *P* < 0.01; ***, *P* < 0.001.

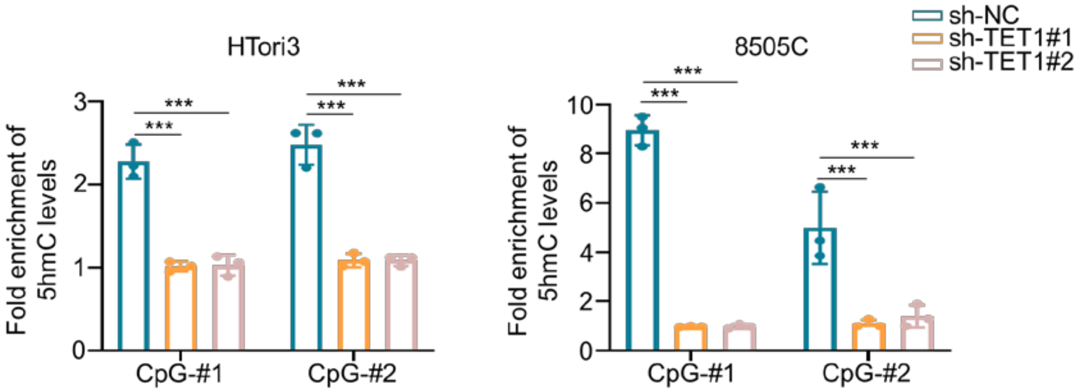

Supplementary Fig. 6 The enrichment of 5hmC in *PAX8* promotor was determined by hMeDIP-qPCR in TET1-knockdown HTori3 and 8505C cells. The data were presented as the mean ± SD. ***, $P < 0.001$.

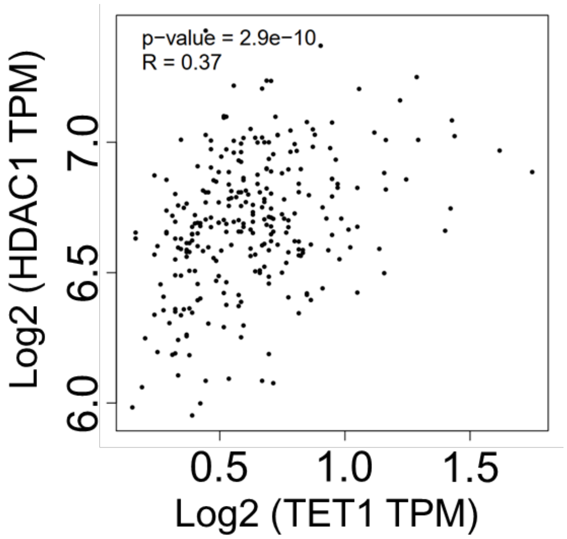

Supplementary Fig. 7 The correlation between *TET1* and *HDAC1* mRNA expression in normal thyroid tissues (Data from GEPIA2 database).

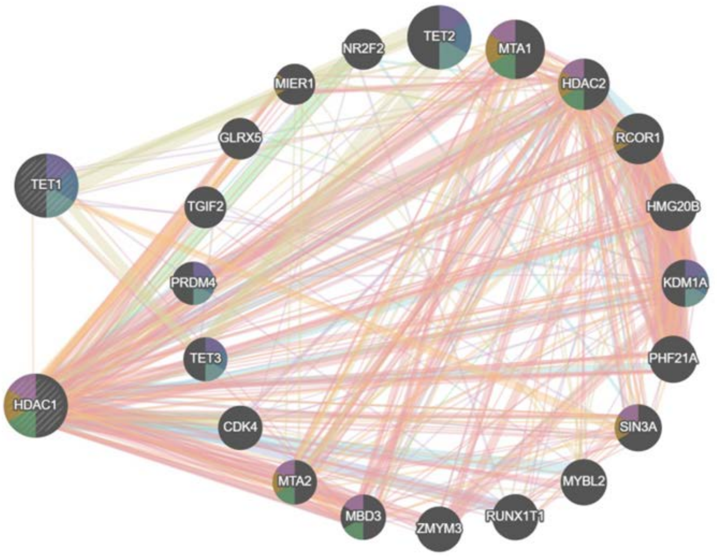

Supplementary Fig. 8 GeneMANIA database was used to predict the interaction between TET1 and HDAC1.

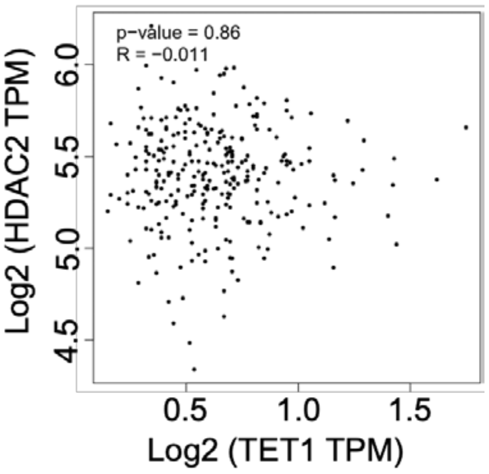

Supplementary Fig. 9 The correlation between *TET1* and *HDAC2* mRNA expression in normal thyroid tissues (Data from GEPIA2 database).

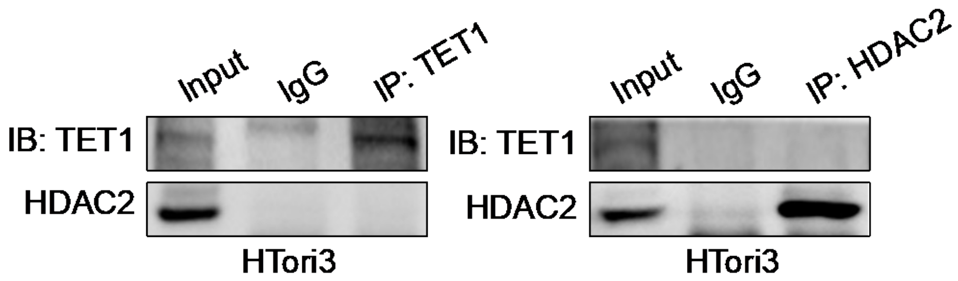

Supplementary Fig. 10 Co-immunoprecipitation (Co-IP) assays demonstrating no interaction between TET1 and HDAC2 in HTori3 cells.

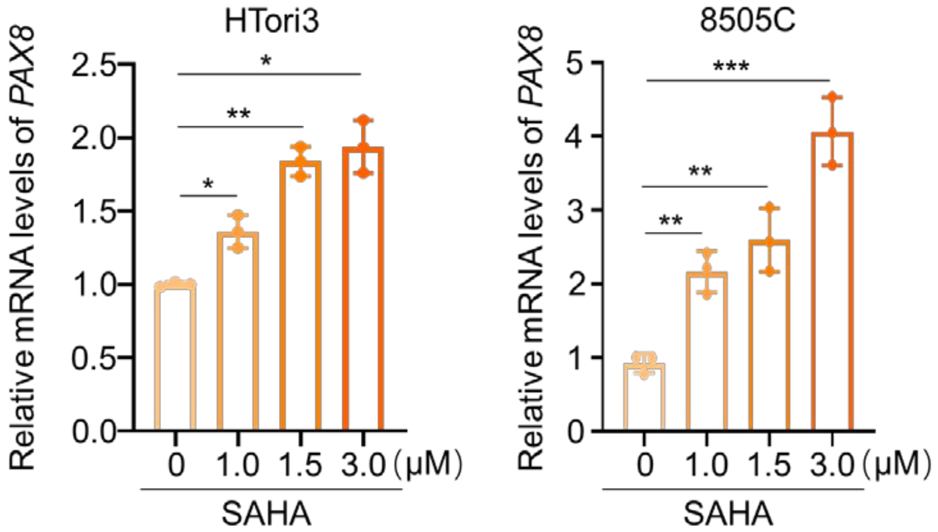

Supplementary Fig. 11 HTori3 and 8505C cells were treated with different doses of vorinostat (SAHA), and mRNA levels of *PAX8* were then determined by qRT-PCR. DMSO was used as the control. The data were presented as the mean ± SD. *, $P < 0.05$; **, $P < 0.01$; ***, $P < 0.001$.

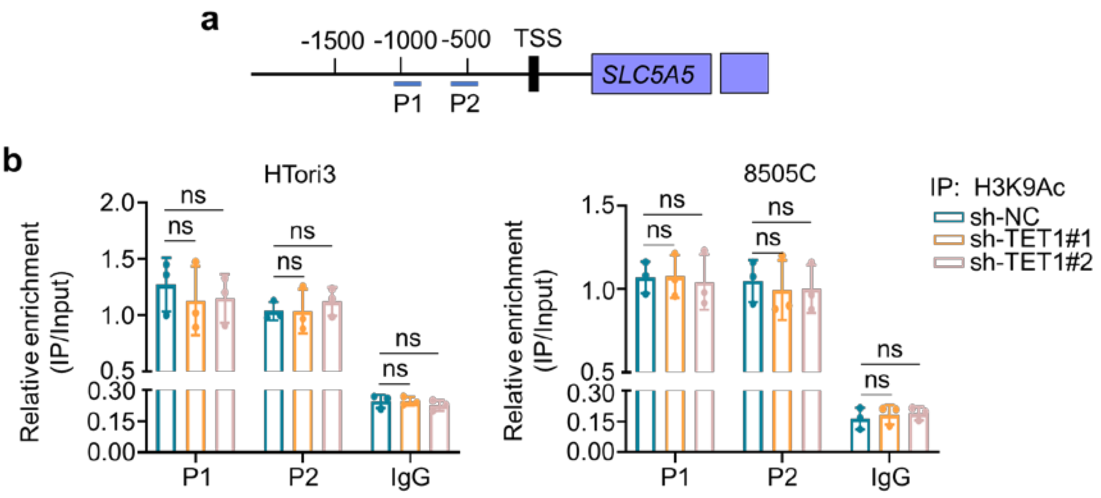

Supplementary Fig. 12 a The primers were designed to amplify two regions (P1 and P2) of the promoter of *SLC5A5*. **b** The enrichment of H3K9Ac in *SLC5A5* promotor was determined by ChIP-qPCR in TET1-knockdown HTori3 and 8505C cells. IgG antibody was used as a negative control. The data were presented as the mean ± SD. ns, no significance.

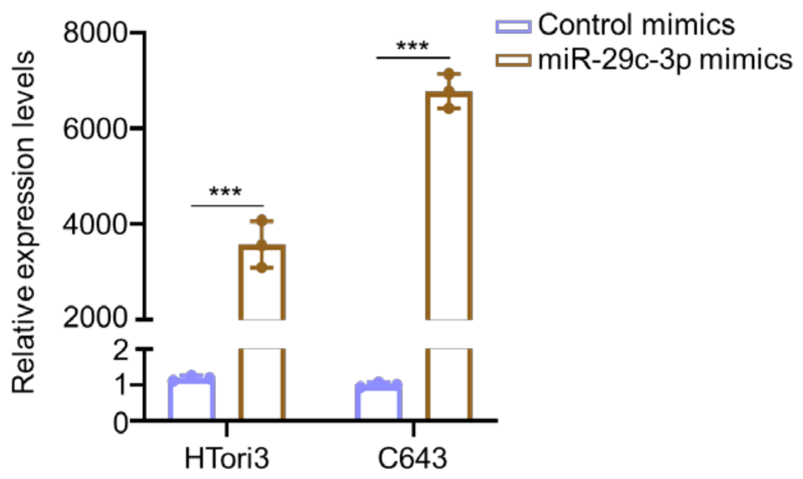

Supplementary Fig. 13 HTori3 and C643 cells were transfected with control mimics and miR-29c-3p mimics, and the levels of miR-29c-3p were then examined by qRT-PCR. *U6* was used as an internal reference. The data were presented as the mean ± SD. ***, *P* <0.001.

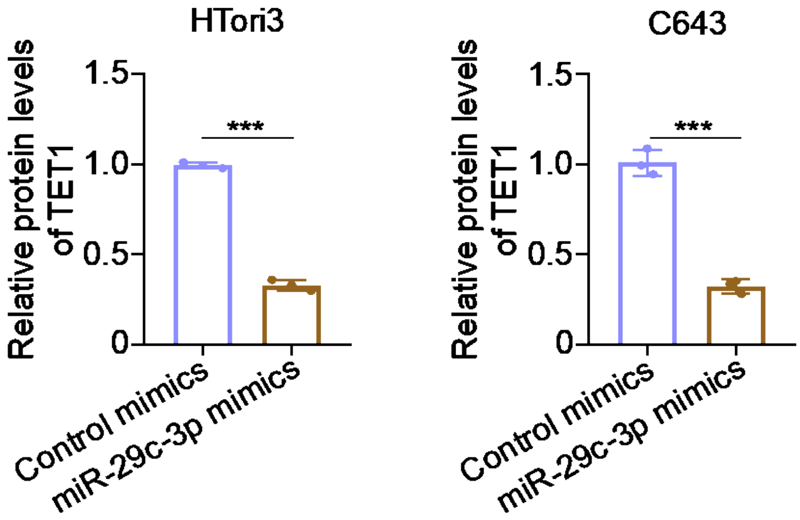

Supplementary Fig. 14 Shown were the quantification of TET1 protein levels relative to β-Actin in HTori3 (left panel) and C643 (right panel) cells (related to Fig. 9h).

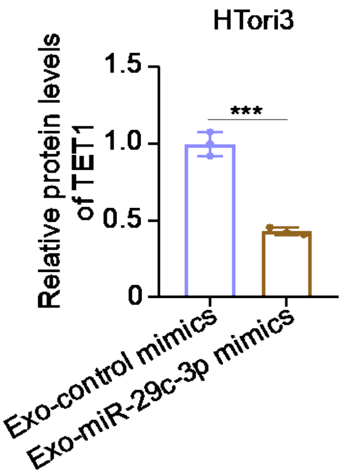

Supplementary Fig. 15 Shown was the quantification of TET1 protein levels relative to β-Actin in HTori3 cells (related to Fig. 9i).
